# Development and retrospective validation of an artificial intelligence system for diagnostic assessment of prostate biopsies: study protocol

**DOI:** 10.1136/bmjopen-2024-097591

**Published:** 2025-07-07

**Authors:** Nita Mulliqi, Anders Blilie, Xiaoyi Ji, Kelvin Szolnoky, Henrik Olsson, Matteo Titus, Geraldine Martinez Gonzalez, Sol Erika Boman, Masi Valkonen, Einar Gudlaugsson, Svein Reidar Kjosavik, José Asenjo, Marcello Gambacorta, Paolo Libretti, Marcin Braun, Radzislaw Kordek, Roman Łowicki, Kristina Hotakainen, Päivi Väre, Bodil Ginnerup Pedersen, Karina Dalsgaard Sørensen, Benedicte Parm Ulhøi, Mattias Rantalainen, Pekka Ruusuvuori, Brett Delahunt, Hemamali Samaratunga, Toyonori Tsuzuki, Emilius Adrianus Maria Janssen, Lars Egevad, Kimmo Kartasalo, Martin Eklund

**Affiliations:** 1 Department of Medical Epidemiology and Biostatistics, Karolinska Institutet, Stockholm, Sweden; 2 Department of Pathology, Stavanger University Hospital, Stavanger, Norway; 3 Faculty of Health Sciences, University of Stavanger, Stavanger, Norway; 4 Department of Molecular Medicine and Surgery, Karolinska Institutet, Stockholm, Sweden; 5 Institute of Biomedicine, University of Turku, Turku, Finland; 6 The General Practice and Care Coordination Research Group, Stavanger University Hospital, Stavanger, Norway; 7 Department of Global Public Health and Primary Care, Faculty of Medicine, University of Bergen, Bergen, Norway; 8 Department of Pathology, SYNLAB, Madrid, Spain; 9 Department of Pathology, SYNLAB, Brescia, Italy; 10 Department of Pathology, Chair of Oncology, Medical University of Lodz, Lodz, Poland; 11 1st Department of Urology, Medical University of Lodz, Lodz, Poland; 12 Department of Clinical Chemistry, University of Helsinki, Helsinki, Finland; 13 Laboratory Services, Mehiläinen Oy, Helsinki, Finland; 14 Mehiläinen Länsi-Pohja Hospital, Kemi, Finland; 15 Department of Radiology, Aarhus University Hospital, Aarhus, Denmark; 16 Department of Clinical Medicine, Aarhus University, Aarhus, Denmark; 17 Department of Molecular Medicine, Aarhus University Hospital, Aarhus, Denmark; 18 Department of Pathology, Aarhus University Hospital, Aarhus, Denmark; 19 InFLAMES Research Flagship, University of Turku, Turku, Finland; 20 Faculty of Medicine and Health Technology, Tampere University, Tampere, Finland; 21 Malaghan Institute of Medical Research, Wellington, New Zealand; 22 Department of Oncology and Pathology, Karolinska Institutet, Stockholm, Sweden; 23 Aquesta Uropathology and University of Queensland, Brisbane, Queensland, Australia; 24 Department of Surgical Pathology, School of Medicine, Aichi Medical University, Nagoya, Japan; 25 Department of Chemistry, Bioscience and Environmental Engineering, University of Stavanger, Stavanger, Norway; 26 Institute for Biomedicine and Glycomics, Griffith University, Brisbane, Queensland, Australia; 27 Department of Medical Epidemiology and Biostatistics, SciLifeLab, Karolinska Institutet, Stockholm, Sweden

**Keywords:** Artificial Intelligence, Prostate, HISTOPATHOLOGY, Retrospective Studies

## Abstract

**Abstract:**

**Introduction:**

Histopathological evaluation of prostate biopsies using the Gleason scoring system is critical for prostate cancer diagnosis and treatment selection. However, grading variability among pathologists can lead to inconsistent assessments, risking inappropriate treatment. Similar challenges complicate the assessment of other prognostic features like cribriform cancer morphology and perineural invasion. Many pathology departments are also facing an increasingly unsustainable workload due to rising prostate cancer incidence and a decreasing pathologist workforce coinciding with increasing requirements for more complex assessments and reporting. Digital pathology and artificial intelligence (AI) algorithms for analysing whole slide images show promise in improving the accuracy and efficiency of histopathological assessments. Studies have demonstrated AI’s capability to diagnose and grade prostate cancer comparably to expert pathologists. However, external validations on diverse data sets have been limited and often show reduced performance. Historically, there have been no well-established guidelines for AI study designs and validation methods. Diagnostic assessments of AI systems often lack preregistered protocols and rigorous external cohort sampling, essential for reliable evidence of their safety and accuracy.

**Methods and analysis:**

This study protocol covers the retrospective validation of an AI system for prostate biopsy assessment. The primary objective of the study is to develop a high-performing and robust AI model for diagnosis and Gleason scoring of prostate cancer in core needle biopsies, and at scale evaluate whether it can generalise to fully external data from independent patients, pathology laboratories and digitalisation platforms. The secondary objectives cover AI performance in estimating cancer extent and detecting cribriform prostate cancer and perineural invasion. This protocol outlines the steps for data collection, predefined partitioning of data cohorts for AI model training and validation, model development and predetermined statistical analyses, ensuring systematic development and comprehensive validation of the system. The protocol adheres to Transparent Reporting of a multivariable prediction model of Individual Prognosis Or Diagnosis+AI (TRIPOD+AI), Protocol Items for External Cohort Evaluation of a Deep Learning System in Cancer Diagnostics (PIECES), Checklist for AI in Medical Imaging (CLAIM) and other relevant best practices.

**Ethics and dissemination:**

Data collection and usage were approved by the respective ethical review boards of each participating clinical laboratory, and centralised anonymised data handling was approved by the Swedish Ethical Review Authority. The study will be conducted in agreement with the Helsinki Declaration. The findings will be disseminated in peer-reviewed publications (open access).

**STRENGTHS AND LIMITATIONS OF THIS STUDY**

- The study protocol incorporates one of the largest datasets of digitised prostate core needle biopsies for the development and retrospective validation of diagnostic artificial intelligence (AI) models.
- The whole slide image data capture a broad spectrum of variation in patient populations, histological sample preparation and scanning instruments across different clinical sites.
- The collection and digitisation of the cohorts has been carefully planned to ensure fully external validation of AI algorithms without information leakage between the data used for AI development and for AI validation.
- We recognise the under-representation of certain demographic groups in these predominantly Caucasian patient cohorts and are committed to addressing this through continued data collection.
- The varying practices and interobserver variation in the reporting of prostate pathology introduce systematic differences in the reference standards across cohorts, which cannot be fully eliminated due to the subjective nature of histopathological assessments.

## Introduction

 Prostate cancer is the second most common malignancy in men globally.^1^ More than two million men undergo prostate biopsy every year in the EU and US alone.^2^ This is further expected to increase markedly in the coming 15 years due to the prolonged life expectancy and widespread adoption of more sensitive screening and diagnostic methods.^3^ Histopathological evaluation of prostate core needle biopsies is crucial for diagnosing and treating prostate cancer. Pathologists examine biopsies using the Gleason scoring system,^4^ assigning primary and secondary grades (eg, Gleason score (GS) of 3+4=7) based on the relative quantities of tissue representing different Gleason patterns.^5^ Grading is, however, inherently subjective and associated with high intrapathologist and interpathologist variability (Cohen’s kappa statistics varying from 0.30 to 0.70 between pathologists), placing patients at risk of inappropriate treatment selection.^6^,^8^ With the aim of standardisation, the International Society of Urological Pathology (ISUP) updated grading guidelines such that GSs are pooled into five ordinal categories (ie, 1–5) referred to as the ISUP grades (also called grade groups or WHO grade).^5 9 10^ Besides Gleason scoring, similar challenges affect the reliable assessment of other histopathological entities relevant to the clinical management of prostate cancer, such as cribriform cancer morphology^11^ or perineural invasion (PNI),^12^ both of which are associated with poor prognosis.

Digital pathology^13^ and the application of artificial intelligence (AI) algorithms to analyse whole slide images (WSIs) hold promise for reducing variability and improving the accuracy of histopathological assessments. Many previous studies have demonstrated that AI can diagnose and grade prostate cancer on par with expert pathologists.^14^,^17^ However, external validations demonstrating the generalisation capacity of these models on data spanning across scanning devices, laboratories and patient populations not involved in the model development have been limited. Moreover, results from the validation studies have often shown deteriorated performance on the external data.^14 18 19^ These complications are not specific to prostate pathology, as there are several examples of scanner-induced variability and bias posing challenges for AI models across different tasks and tissue types.^20^,^22^

The unresolved issues with generalisation limit the widespread application of AI in clinical practice, including histopathology. The field has historically lacked well-established guidelines on AI study designs and standardised methods for the proper evaluation and reporting of AI validation studies. Generally, diagnostic assessments of AI systems lack preregistered study protocols with predefined analysis plans and rigorous sampling of external cohorts, which are key factors for generating reliable evidence of the safety and diagnostic accuracy of these systems in view of further prospective evaluations in clinical trials.^23 24^ Here, we present a comprehensive study protocol for the development and retrospective validation of an AI system for diagnostic assessment of prostate biopsies. This protocol outlines study objectives, analysis and experimental pipelines, as well as data cohorts for evaluating the generalisability and robustness of the AI system. The AI system is ultimately intended to be used as part of computer-aided diagnosis software to provide decision-making support for pathologists. However, this study focuses solely on the standalone diagnostic performance of the system, excluding clinical implementation, user interaction and combined analysis with human pathologist supervision aspects.

Several guidelines have recently been proposed or are under development for reporting clinical validation studies of AI-based methods, for example, SPIRIT-AI (Standard Protocol Items: Recommendations for Interventional Trials-AI) and its companion statement CONSORT-AI (Consolidated Standards of Reporting Trials-AI), which are intended for protocols and reporting of randomised clinical trials involving an AI intervention component,^25 26^ or the DECIDE-AI (Developmental and Exploratory Clinical Investigations of DEcision support systems driven by Artificial Intelligence) guideline which applies specifically to early, small-scale evaluation of AI interventions, with a focus on clinical utility, safety and human factors.^27^ In terms of guidelines applicable to preclinical and offline evaluation of AI prediction models, the TRIPOD+AI (Transparent Reporting of a multivariable prediction model of Individual Prognosis Or Diagnosis+AI)^28^ guideline on developing or reporting the performance of AI prediction models has recently been released,^29^ while the STARD-AI (Standards for Reporting of Diagnostic Accuracy Study-AI)^30^ guideline is still under development. This protocol incorporates guidelines by the TRIPOD+AI,^29^ applicable parts of the best practice checklists proposed in PIECES (Protocol Items for External Cohort Evaluation of a Deep Learning System in Cancer Diagnostics),^31^ CLAIM (Checklist for AI in Medical Imaging)^32 33^ and other methodological checklists, including those for radiology due to the lack of similar guidelines in pathology.^34^

To our knowledge, the studies outlined in this protocol represent the largest retrospective validation of an AI system for prostate cancer diagnosis using digitised core needle biopsies. Although all data cohorts and partitions are predefined, the protocol is designed to allow the addition of new validation cohorts without altering the initial partitions. Thus, this protocol will be extended as needed to support further retrospective validation of the AI system on other patient populations on a global scale. Furthermore, in addition to its current diagnostic applications, the AI system will serve as a foundation for future systems aimed at prognostication, treatment response prediction and reducing reliance on immunohistochemistry (IHC). A prospective clinical trial, to be described in a separate protocol, is planned to evaluate the real-world performance of the AI system and its integration into clinical workflows.

## Methods and analysis

### Study objectives

The objective of the study is to develop a high-performing and robust AI model for diagnosis and Gleason scoring of prostate cancer in core needle biopsies, and at scale demonstrate that it can generalise to fully external data from independent patients, pathology laboratories and digitalisation platforms.

#### Primary objective

The primary objective is to assess the concordance between the AI model and pathologists in diagnosing and Gleason-scoring prostate cancer in core needle biopsies.

#### Secondary objectives

There are three secondary objectives which this study accommodates:

- Assess the concordance between the AI model and pathologists in measuring cancer extent (in millimetres) in prostate core needle biopsies.
- Assess the concordance between the AI model and pathologists in detecting PNI in prostate core needle biopsies.
- Assess the concordance between the AI model and pathologists in detecting cribriform cancer in prostate core needle biopsies.

### AI system

The AI system developed and validated in this study is intended for the histopathological assessment of digitised prostate core needle biopsies. The system will be based on deep neural networks and its specific design (eg, image preprocessing steps, model architecture and training approach) will be optimised during the study (see online supplemental appendix 1 for further description of the design choices and hyperparameters that will be evaluated). This study comprises multiple AI models, each tailored for the specific objectives, that is, grading, PNI, cribriform cancer and cancer length, and together these models integrate to form an AI system.

#### System input

A WSI stored in a supported vendor-specific format, depicting a formalin-fixed, paraffin-embedded haematoxylin & eosin (H&E) stained prostate core needle biopsy specimen with one or several tissue cuts of one or several biopsy cores.

#### System output

- GS: the system will output GS, such as 4+3=7, indicating the primary and secondary patterns observed within the input WSI. The GS ranges from 3+3=6 to 5+5=10, with lower scores representing less aggressive cancer and higher scores indicating more aggressive cancer. Benign samples are encoded as 0+0.
- ISUP grade: the system will output an ISUP grade which groups GS into ordinal categories, ranging from 1 to 5. The GS are expressed as ISUP grades as follows: ISUP 1 (GS 6), ISUP 2 (GS 3+4=7), ISUP 3 (GS 4+3=7), ISUP 4 (GS 8), ISUP 5 (GS 9–10). Benign samples are encoded as 0.
- Cancer extent: the system will quantify the extent of cancer within the provided WSI in millimetres. This measurement indicates the size of the cancerous area within the tissue specimen.
- Cribriform cancer: the system will output the predicted probability of cribriform prostate cancer morphology being present within the input WSI.
- PNI: the system will output the predicted probability of PNI being present within the input WSI.
- Visualisation: the system will provide a visualisation of its predictions including areas of different Gleason patterns, PNI and cribriform cancer, which can be examined in a WSI viewer software overlaid on the digital slide. The exact format of the visualisation will vary depending on the viewer software.

### Study design

**Figure 1:**
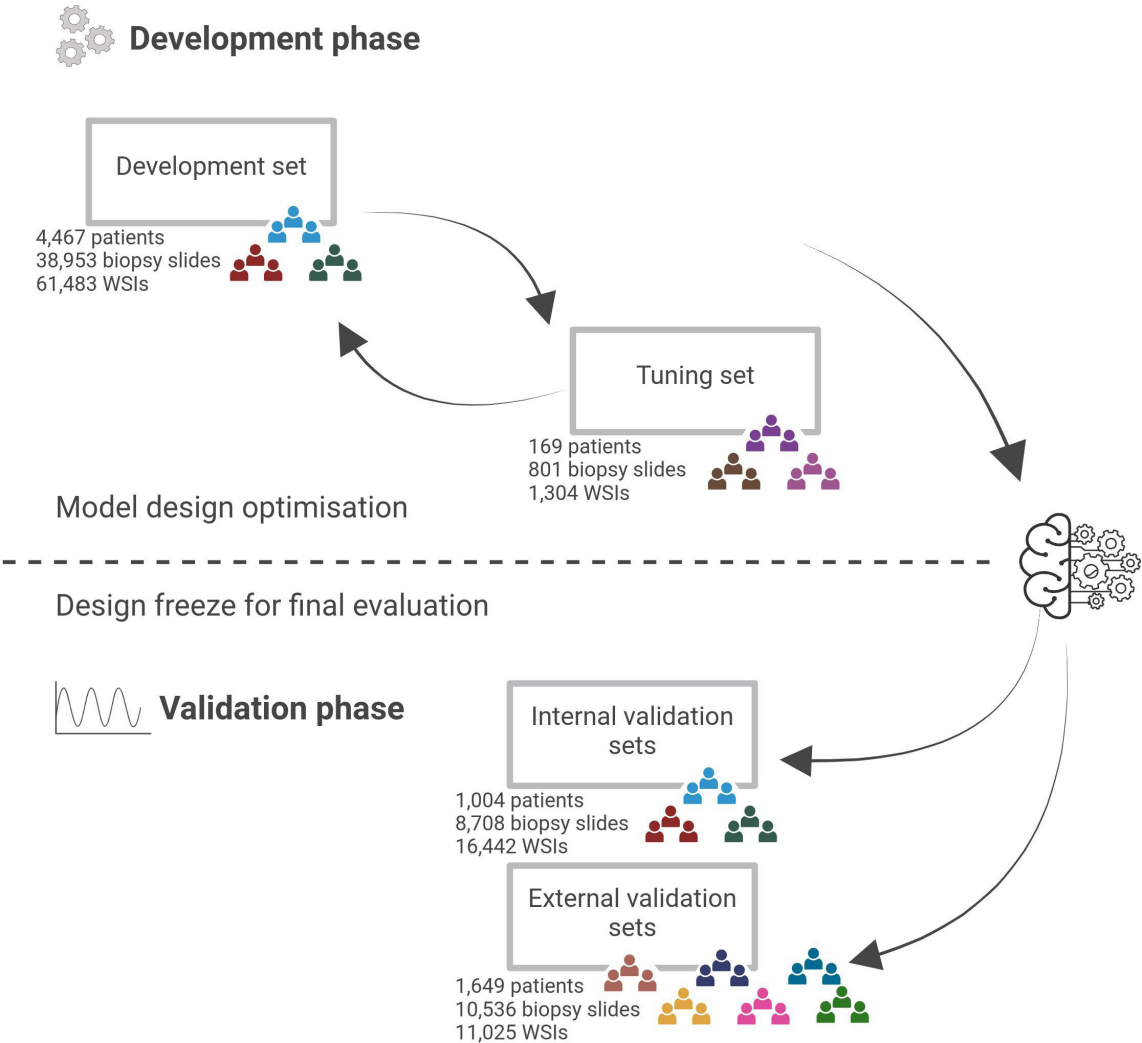
Overview of the study design. The study design has two main steps: (top) The development phase involves model design optimisation through an iterative process of experiments. In each experiment, the model is trained and its performance is evaluated on the development set using cross-validation and on a separate tuning set. (bottom) The validation phase is initiated with a design freeze, after which no further changes to the model take place. Validation comprises the assessment on the internal data (ie, collected from the same laboratory and/or using the same scanner as development data) and the external data (ie, collected from other laboratories using other scanners than any of the development data). The colour-coding of the cohort icons represents the shared origin of data used for development and internal validation, and the fully independent nature of the external validation and tuning cohorts. Created in BioRender. Mulliqi, N. (2025) https://BioRender.com/evzu6sn. WSIs, whole slide images.

The aim of this study is to develop the AI system described above and validate its diagnostic performance on retrospectively collected cohorts. To carry out the study, historical data, including medical records, pathology reports and digitised images, have been collected for cases where both the AI system and human pathologists make diagnostic assessments. The study design involves two independent phases: AI system development and AI system validation as shown in figure 1. The development phase involves an iterative cycle of refining the model design and hyperparameters using predefined development and tuning cohorts for model training and estimation of the effects of design choices on diagnostic performance. Once the overall performance on the development and tuning sets is deemed to have reached a plateau and further changes to the model design no longer yield meaningful improvements, a design freeze will take place and the final AI model will be graduated to the validation phase. This design achieves complete isolation between the model development and the retrospective validation to avoid any information leakage, which could lead to overly optimistic validation results. All model parameters and hyperparameters, including selection of any classifier thresholds, will be set based on the development and tuning cohorts, and no adjustments or tweaking will be conducted on the validation cohorts, which will remain entirely untouched during the development phase. Development data, on the other hand, can be freely accessed without risk of bias at any point, allowing optimisation of the AI model design prior to the validation phase of the study.

The development cohorts provide a wide representation of tissue morphologies, scanning devices, laboratories and clinical characteristics of patients, allowing for the training of a robust model. The tuning cohorts enable assessing model generalisation (ie, performance on data from different laboratories and scanners than the development cohorts) on each development iteration, and direct performance comparison with state-of-the-art models evaluated on these same datasets in earlier studies.^15 17^ Sequential experiments will be conducted one modification at a time to evaluate, for example, different preprocessing approaches for extracting image data from the WSI, deep neural network architectures, optimiser hyperparameters, etc (see online supplemental appendix 1). Model performance at each step will be measured using cross-validation on the development cohorts and independent evaluation on the tuning cohorts.

The validation phase will employ a blinded approach, wherein neither the pathologists nor the AI model have access to each other’s assessments. The validation cohorts consist of samples representing a range of heterogeneous clinical settings and were collected from patients not included in the development or tuning cohorts. They are categorised as internal (scanner and laboratory included in the development set), partly external (scanner included in the development set) or fully external (neither scanner nor laboratory included in the development set) depending on the slide scanners and clinical laboratories involved. Internal validation can be expected to provide an optimistic estimate of the diagnostic performance of the AI model in the absence of laboratory or scanner variation. The generalisation performance of the model will ultimately be evaluated on the external validation cohorts, which avoids any optimistic bias. The design also allows for additional validation cohorts to be added at any point after the development phase.

Due to interobserver variability among pathologists, reference standards established by pathologists vary across different validation cohorts. This complicates the assessment of the AI model for generalisation across cohorts, as any differences in observed performance can be partly attributed to differences in reference standards and partly attributed to imperfect AI generalisation to data originating from different clinical sites. In the case of the primary study objective of Gleason scoring, we have addressed this issue by having a representative subset of slides from each cohort be reassessed by the lead pathologist (LE). The lead pathologist is highly experienced in urological pathology and has shown high concordance relative to other experienced uropathologists in several studies.^17 35 36^ For the secondary study objectives of cribriform cancer and PNI detection, the assessments were conducted either by the lead pathologist or by other experienced (>25 years of clinical experience after residency) uropathologists (BD, HS) whose concordance with the lead pathologist has been quantified in earlier studies.^11 12^ This provides a consistent reference standard which will allow us to assess the technical generalisation performance of the model (without complete confounding between laboratory, scanner and pathologist reference standards), in addition to large-scale evaluation relying on the varying reference standards provided by the local pathologists for each cohort.

Clinical and pathological characteristics of the included patients are summarised in online supplemental appendix 2, and detailed information on the slide scanning is provided in table 1. Details on reference standards for each cohort with respect to grading are given in table 2, and with respect to cribriform cancer and PNI are given in table 3. Information on slides representing morphological subtypes is given in table 4, and the number of slides for which IHC staining was performed in order to confirm the diagnosis is tabulated in table 5. Online supplemental appendix 2 shows CONSORT diagrams and a comprehensive summary of the data cohorts (including patient characteristics and selection, biopsy acquisition, histopathological sample preparation, digitisation and reference standard protocols). Due to variations in reporting practices and availability of detailed clinical information across the included sites and datasets, not all clinical characteristics or scanning metadata are available for all patient cohorts (missing data are indicated as N/A in the corresponding tables).

**Table 1:** Overview of image acquisition attributes and WSIs

Split	Cohort	Scanning location	Scanning period	Scanner			Magnification (pixel size)	WSI format	WSI number
				Vendor	Model	Serial no.			
Development, tuning and internal validation cohorts	STHLM3*	Department of Medical Epidemiology and Biostatistics, Karolinska Institutet, Solna, Sweden	July 2014–November 2014	Hamamatsu	NanoZoomer 2.0-HT C9600-12	760 347	20× (0.4520 µm)	.ndpi	5726
								.tiff	3417
		SciLifeLab, Uppsala, Sweden	September 2017–June 2019	Aperio	AT2 DX	RUD-D10971	20× (0.5032 µm)	.svs	3667
								.tiff	2445
		Department of Medical Epidemiology and Biostatistics, Karolinska Institutet, Solna, Sweden	March 2018–June 2019	Hamamatsu	NanoZoomer XR C12000-02	870 003	20× (0.4536 µm)	.ndpi	17 973
		Department of Medical Epidemiology and Biostatistics, Karolinska Institutet, Solna, Sweden	October 2019–June 2020	Philips	IntelliSite UFS	FMT0047	40× (0.2500 µm)	.isyntax	32 078
		Department of Medical Epidemiology and Biostatistics, Karolinska Institutet, Solna, Sweden	February 2023–March 2023	Grundium	Ocus40	MGU-00 003–0 00 184	40× (0.2505 µm)	.svs	2289
	SUH	Department of Pathology, Stavanger University Hospital, Stavanger, Norway	February 2022–March 2023	Hamamatsu	NanoZoomer S60 C13210-01	000266	40× (0.2199 µm)	.ndpi	5762
	RUMC	Radboud University Medical Center, Nijmegen, The Netherlands	January 2019–December 2019	3DHISTECH	Pannoramic Scan ll	N/A	20× (0.4861 µm)	.tiff	5275
	STG*	Department of Immunology, Genetics, and Pathology, Uppsala University, Uppsala, Sweden	September 2018–October 2018	Hamamatsu	C13210	000058	20× (0.4405 µm)	.ndpi	74
		Department of Immunology, Genetics, and Pathology, Uppsala University, Uppsala, Sweden	October 2018	Hamamatsu	C13210	000044	20× (0.4409 µm)	.ndpi	67
		SciLifeLab, Uppsala, Sweden	December 2018	Aperio	AT2 DX	RUD-D10971	20× (0.5032 µm)	.svs	247
	KUH-1	Department of Pathology, Karolinska University Hospital, Solna, Sweden	July 2019–August 2019	Hamamatsu	NanoZoomer S360 C13220-01	000077	20× (0.4604 µm)	.ndpi	330
								.tiff	330
External and partly external validation cohorts	AMU	Aichi Medical University, Nagakute, Japan	January 2023–December 2023	Hamamatsu	C13210	000218	40× (0.2211 µm)	.ndpi	73
	AQ	Department of Medical Epidemiology and Biostatistics, Karolinska Institutet, Solna, Sweden	October 2019–June 2020	Philips	IntelliSite UFS	FMT0047	40× (0.2500 µm)	.isyntax	58
		Department of Medical Epidemiology and Biostatistics, Karolinska Institutet, Solna, Sweden	January 2024–February 2024	Grundium	Ocus40	MGU-00 003–0 00 184	40× (0.2505 µm)	.svs	78
	AUH	Department of Pathology, Aarhus University Hospital, Aarhus, Denmark	November 2019–June 2020	Hamamatsu	NanoZoomer 2.0-HT C9600-12	1Z0209	20× (0.4545 µm)	.ndpi	102
	KUH-2	Department of Pathology, Karolinska University Hospital, Solna, Sweden	July 2022	Aperio	AT2 DX	SS7033	20× (0.5032 µm)	.svs	146
	MLP	Finnish Institute of Molecular Medicine, Helsinki, Finland	October 2019–March 2020	3DHISTECH	Pannoramic 250 Flash III	01702	40× (0.2427 µm)	.mrxs	1964
	MUL*	Department of Medical Epidemiology and Biostatistics, Karolinska Institutet, Solna, Sweden	December 2019–January 2020	Philips	IntelliSite UFS	FMT0047	40× (0.2500 µm)	.isyntax	503
		Department of Medical Epidemiology and Biostatistics, Karolinska Institutet, Solna, Sweden	January 2023–March 2023	Grundium	Ocus40	MGU-00 003–0 00 184	40× (0.2505 µm)	.svs	1945
	SCH & SFI & SFR	Synlab Italia srl, Monza, Italy	June 2022–February 2023	Philips	IntelliSite UFS	N/A	40× (0.2500 µm)	.isyntax	3486
	SPROB20	Uppsala University Hospital, Uppsala, Sweden	2020	Hamamatsu	NanoZoomer S360 C13210	N/A	40× (0.2204 µm)	.tif	2570
	UKK	Institute of Pathology, University Hospital Cologne, Cologne, Germany	N/A	Hamamatsu	NanoZoomer S360	N/A	40× (0.2305 µm)	.ome.tiff	50
	WNS	Hospital Wiener Neustadt, Wiener Neustadt, Austria	N/A	Hamamatsu	NanoZoomer S360	N/A	40× (0.2305 µm)	.ome.tiff	50

Cohorts marked with (*) (ie, STHLM3, STG and MUL) contain overlapping subsets of slides digitised with different scanners. Other cohorts were either digitised with a single scanner or contain non-overlapping subsets of slides digitised with different scanners.

AMU, Aichi Medical University; AQ, Aquesta Uropathology; AUH, Aarhus University Hospital; KUH-1, Karolinska University Hospital; KUH-2, KUH morphological subtypes; MLP, Mehiläinen Länsi-Pohja; MUL, Medical University of Lodz; N/A, not available; RUMC, Radboud University Medical Center; SCH, Synlab Switzerland; SFI, Synlab Finland; SFR, Synlab France; SPROB20, Spear Prostate Biopsy 2020; STG, Capio S:t Göran Hospital; STHLM3, Stockholm3; SUH, Stavanger University Hospital; UKK, University Hospital Cologne; WNS, Hospital Wiener Neustadt; WSI, whole slide image.

**Table 2:** Reference standard protocols with respect to grading

Cohorts				Reference standard protocol			
Split	Cohort	Cohort subset	Slide number	Type	Total number of readers	Level	
Development, tuning and internal validation cohorts	STHLM3	STHLM3 full cohort	36 848	Single reader (LE)	1	Slide	Patient
		ImageBase	90	Panel	23	Slide	
		PANDA Swedish private validation set	212	Consensus	3		
		STHLM3 morphological subtypes	24	Single reader (LE)	1		
	SUH	SUH full cohort	5762	Single reader	14	Slide	
		Re-graded	66	Single reader (LE)	1		
	RUMC	RUMC full cohort	5275	Single reader	Multiple	Slide	
		PANDA RUMC tuning set	195	Panel	3		
		PANDA RUMC private validation set	333	Panel	3		
		Re-graded	66	Single reader (LE)	1		
	STG	STG full cohort	247	Single reader (LE)	1	Slide	
	KUH-1	KUH-1 full cohort	330	Single reader (LE)	1	Slide	Patient
External and partly external validation cohorts	AMU	AMU full cohort	73	Single reader	1	Patient	
	AQ	AQ full cohort	136	Single reader	1	Slide	
	AUH	AUH full cohort	102	Single reader	1	Slide	
		Re-graded	41	Single reader (LE)	1		
	KUH-2	KUH-2 full cohort	146	Single reader (LE)	1	Slide	
	MLP	MLP full cohort	1964	Single reader	Multiple	Location	
		Re-graded	66	Single reader (LE)	1	Slide	
	MUL	MUL full cohort	1959	Consensus	2	Slide	
		Re-graded	66	Single reader (LE)	1		
	SCH	SCH full cohort	2434	Single reader	Multiple	Location	
		Re-graded	72	Single reader (LE)	1	Slide	
	SFI	SFI full cohort	537	Single reader	Multiple	Location	
		Re-graded	67	Single reader (LE)	1	Slide	
	SFR	SFR full cohort	515	Single reader	Multiple	Location	
		Re-graded	49	Single reader (LE)	1	Slide	
	SPROB20	SPROB20 full cohort	2570	Single reader	Multiple	Patient	
		Re-graded	50	Single reader (LE)	1	Slide	
	UKK	UKK full cohort	50	Panel	11	Slide	
	WNS	WNS full cohort	50	Panel	10	Slide	

Reference standard protocols are divided into three categories: single reader, consensus and panel. In the single reader category, a sole reader assessed each slide. In the consensus category, assessments from multiple readers were combined based on site-specific criteria for consensus. In the panel category, readers provided independent assessments in a blinded manner.

AMU, Aichi Medical University; AQ, Aquesta Uropathology; AUH, Aarhus University Hospital; KUH-1, Karolinska University Hospital; KUH-2, KUH morphological subtypes; MLP, Mehiläinen Länsi-Pohja; MUL, Medical University of Lodz; RUMC, Radboud University Medical Center; SCH, Synlab Switzerland; SFI, Synlab Finland; SFR, Synlab France; SPROB20, Spear Prostate Biopsy 2020; STG, Capio S:t Göran Hospital; STHLM3, Stockholm3; SUH, Stavanger University Hospital; UKK, University Hospital Cologne; WNS, Hospital Wiener Neustadt.

**Table 3:** Reference standard protocols with respect to PNI and cribriform cancer

Cohorts				Reference standard protocol			
Split	Cohort	Cohort subset	Slide number	Type	Total number of readers	Level	
Development, tuning and internal validation cohorts	STHLM3	STHLM3 full cohort	36 848	Single reader (LE)	1	Slide	Patient
		Re-assessed cribriform cancer (round 1)	702	Single reader (LE)	1	Slide	Pixel
		Re-assessed cribriform cancer (round 2)	304	Panel	9	Slide	
		Re-assessed PNI (round 1)	485	Single reader (LE)	1	Slide	Pixel
		Re-assessed PNI (round 2)	212	Panel	4	Slide	
	SUH	SUH full cohort	N/A	N/A	N/A	N/A	
		Re-assessed cribriform cancer (round 1)	332	Single reader (AB)	1	Slide	
		Re-assessed cribriform cancer (round 2)	200	Single reader (LE)	1	Slide	
		Re-assessed PNI (round 1)	509	Single reader (AB)	1	Slide	
		Re-assessed PNI (round 2)	185	Single reader (LE)	1	Slide	
External validation cohorts	AMU	AMU full cohort	73	Single reader	1	Slide	
	MUL	MUL full cohort	N/A	N/A	N/A	N/A	
		Re-assessed cribriform cancer	276	Consensus	2	Slide	
		Re-assessed PNI	276	Consensus	2	Slide	
	SCH	SCH full cohort	N/A	N/A	N/A	N/A	
		Re-assessed cribriform cancer	56	Single reader (HS)	1	Slide	
		Re-assessed PNI	94	Single reader (BD)	1	Slide	

Reference standard protocols are divided into three categories: single reader, consensus and panel. In the single reader category, a sole reader assessed each slide. In the consensus category, assessments from multiple readers were combined based on site-specific criteria for consensus. In the panel category, readers provided independent assessments in a blinded manner. The SUH, MUL and SCH cohorts do not have consistent original reporting on cribriform cancer and PNI.

AMU, Aichi Medical University; MUL, Medical University of Lodz; N/A, not available; PNI, perineural invasion; SCH, Synlab Switzerland; STHLM3, Stockholm3; SUH, Stavanger University Hospital; WSI, whole slide image.

**Table 4:** Summary of slides representing various morphological subtypes

Morphological subtype	Internal validation cohort	Partly external validation cohort	External validation cohort	
	STHLM3 (n=24)	AQ (n=58)	AQ (n=78)	KUH-2 (n=146)
Adenosis	4	18	7	34
Atrophy	0	0	0	38
Partial atrophy	0	7	9	0
Simple atrophy	0	1	15	0
Basal cell hyperplasia	0	10	7	20
Cancer of atrophic type	7	1	3	2
Clear cell cribriform hyperplasia	0	0	5	3
Cowper’s glands	0	2	14	6
Foamy gland cancer	0	4	2	13
Increased number of glands	0	0	5	0
Postatrophic hyperplasia	0	2	2	4
Prostatectomy*	0	1	4	0
PIN-like cancer	3	0	0	0
Pseudohyperplastic cancer	9	4	0	24
Sclerosing adenosis	0	4	2	0
Seminal vesicle	0	5	7	0
Small cell cancer	0	0	0	4
TUR-P*	0	13	4	0

The AQ cohort contains partly external validation data (scanner was used in the development) and fully external validation data (scanner was not used in the development). A single slide can be associated with multiple subtypes. Instead of morphological subtypes, the samples denoted with (*) represent other types of specimens than core needle biopsies. Besides assessing performance on unusual and potentially challenging morphologies, we will assess how the AI system intended for needle biopsies will respond to other specimen types and evaluate frameworks for automatically flagging outlier cases.^39^

AI, artificial intelligence; AQ, Aquesta Uropathology; KUH-2, Karolinska University Hospital morphological subtypes; PIN, prostatic intraepithelial neoplasia; STHLM3, Stockholm3; TUR-P, transurethral resection of the prostate.

**Table 5:** Summary of slides with IHC staining confirming the diagnosis

Split	Cohort	IHC performed	Number of slides						
			All	Benign	ISUP 1 (3+3)	ISUP 2 (3+4)	ISUP 3 (4+3)	ISUP 4 (4+4, 3+5, 5+3)	ISUP 5 (4+5, 5+4, 5+5)
Internal validation	SUH	Yes	247	132	60	16	10	9	20
		No	909	604	93	60	64	44	44
External validation	SCH	Yes	365	120	131	47	46	9	12
		No	2064	1455	194	154	137	85	35
		Missing	5	5	0	0	0	0	0
	SFR	Yes	116	66	41	4	1	1	0
		No	398	306	46	24	2	9	6
		Missing	1	1	0	0	0	0	0

The number of slides stratified by ISUP grade with/without IHC staining performed to confirm the diagnosis. For the SCH and SFR cohorts, where pathology reporting was performed on anatomical location or patient level, the total summed numbers of slides associated with an IHC-supported diagnosis are shown.

IHC, immunohistochemistry; ISUP, International Society of Urological Pathology; SCH, Synlab Switzerland; SFR, Synlab France; SUH, Stavanger University Hospital.

### Inclusion and exclusion criteria

Provided below are the detailed criteria used to assess the eligibility of patients, individual biopsy slides or WSIs for inclusion in this study.

#### Inclusion criterion

Patients who underwent a prostate core needle biopsy were eligible.

#### Exclusion criteria

- Clinical information:
  - Patients with either slides or associated pathology information unavailable.
  - Slides lacking identifiers (IDs) preventing linkage to the pathology data.
  - Slides with identical IDs preventing unambiguous linkage to the pathology data.
  - Slides with mismatching GS and ISUP grade information.
  - Slides with mismatching information concerning malignancy and GS or ISUP grade (eg, indicated benign but a GS is provided).
  - Slides with partial or erroneous GS reporting (eg, <6, 4+0 or 1+1, etc).
- Staining and slide preparation:
  - Samples not containing prostate tissue, for example, bladder biopsies, testicular biopsies.
  - Samples not stained with H&E (eg, IHC stains).
  - Initial cuts of tissue blocks deemed unsuitable by the pathologist for providing a diagnosis and requiring a recut.
  - Empty biopsy slides with no tissue on the glass.
- Slide integrity and annotation:
  - Slides with pen mark annotations that cover a vast amount of the tissue, obscuring the underlying morphology.
  - Slides with pen mark annotations conflicting with the pathology diagnosis (eg, there exists a pen mark annotation on the slide, but the slide is diagnosed as benign or vice versa). This only applies to the STHLM3 cohort, where the pen mark annotation process is known to be consistent for all samples (see Stockholm3 (STHLM3) in online supplemental appendix 2).
  - Slides with pen mark annotations that result in the majority of the tissue being out of focus during scanning.
- Slide digitisation:
  - Earlier scans of the same slide on the same scanner instrument, assuming the latest WSI represents a successful rescanning due to, for example, earlier focus issues.
  - Corrupt WSI files which cannot be accessed with OpenSlide^37^ or OpenPhi.^38^

### Data partitions

#### Requirements for data partition

We established a number of requirements to guide the inclusion, exclusion and partitioning of data into development, tuning and validation sets to account for several sources of potential bias in the training and validation of the model. We followed available guidelines and criteria for balanced and representative data partitions^32 34 39 40^ and arrived at the following set of requirements:

1. Representative sample selection: Ensure the data are representative of the diversity encountered in clinical practice by including multisite cohorts with variations in scanning equipment (eg, vendors, models, image formats), biopsy preparation (eg, staining, tissue cutting), morphological heterogeneity (eg, different GSs and rare cancer subtypes) and patient demographics.
2. Representative sample size: Include a sufficiently large sample for development and validation to increase the probability of generalisability in the larger population.
3. Mitigate overfitting due to observer bias: Alleviate the possibility of overfitting or ‘over tweaking’ of the model, which may be caused by excessive refinement of the model design aimed at maximising cross-validation performance in development data, since that can jeopardise generalisation outside the development cohorts. The issue can be mitigated by additional (external) tuning data cohorts serving as a less biased performance indicator during model development. It should be further ensured that the tuning cohorts are independent of model training (eg, criteria for early stopping of model training should be assessed only on the development data).
4. Ensure independence of specimens between data partitions: Each data partition (development, tuning, internal or external validation sets) should be independent of the others with no overlap of biopsies or patients.
5. Ensure independence of the sample preparation process between data partitions: Sample external cohorts such that there is no overlap with respect to the clinical laboratories that prepared these cohorts and the development cohorts.
6. Ensure independence of the digitisation process between data partitions: Sample external cohorts such that there is no overlap with respect to the scanning device used for these cohorts and the development cohorts.

#### Predefined data partitions

The process of splitting the data cohorts into development, tuning, and internal and external validation sets was conducted adhering to the requirements for data partitions and is described below (see figure 1 for an overview). The characteristics of the data cohorts included in this study are described in detail in online supplemental appendix 2.

The development set was sampled from the following cohorts: Capio S:t Göran Hospital (STG), Radboud University Medical Center (RUMC), Stavanger University Hospital (SUH) and Stockholm3 (STHLM3). From the RUMC, STHLM3 and SUH cohorts, the patients who were not allocated to tuning or validation sets (see below) were assigned to the development set (approximately 80% of patients). Given the limited size and skewed grade distribution of the STG cohort, it was fully allocated into the development set. The development set covers several clinical laboratories and scanner devices as well as a large degree of variation in tissue morphology and the clinical characteristics of patients, in part due to the largest cohort, STHLM3, originating from a population-based screening trial (Requirements 1–2). Each of the development cohorts was further split into 10 cross-validation folds by randomly allocating patients to folds, stratified by the maximum slide level ISUP grade of each patient.

The tuning set was sampled from the following cohorts: Karolinska University Hospital (KUH-1), RUMC and STHLM3. The entire KUH-1 cohort was assigned to tuning and represents a fully external cohort relative to the development set (ie, different patients, laboratory and scanner). This set also corresponds to the European external validation cohort of the PANDA challenge.^17^ The subsets of the RUMC and STHLM3 cohorts assigned to the tuning set represent internal data relative to the development set (ie, different patients but the same laboratories and scanners) and correspond to the PANDA public test sets in Kaggle (ie, the PANDA tuning sets). The tuning sets allow for evaluating the effects of model design changes on data that is independent of the development set, direct comparison with state-of-the-art models from PANDA, and in the case of KUH-1, assessing the generalisation performance of the model prior to design freeze (ie, performance on data coming from different patients, laboratories and scanners compared with the development data) (Requirement 3). A subset of slides belonging to the PANDA Swedish tuning set was allocated to the internal validation set for reasons related to patient stratification and the inclusion of specific subsets of interest in the internal validation (see below).

The internal validation set was sampled from the following cohorts: RUMC, STHLM3 and SUH, consisting of patients who were not part of the development or tuning sets but whose biopsies were obtained from the same clinical laboratories and scanned with the same scanners as the development and tuning set samples. The STHLM3 internal validation set includes the following subsets, supplemented with randomly sampled patients to achieve a total 20% fraction of patients assigned to tuning and validation: ImageBase,^36^ Swedish private test set in Kaggle (ie, PANDA Swedish internal validation set),^17^ PNI multiobserver validation set^41^ and rare morphological subtypes set.^42^ Including these samples as subsets of the internal validation set will facilitate (internal) comparisons with results obtained in the papers referenced in the preceding sentence. The SUH internal validation set includes the following subsets, supplemented with randomly sampled patients to achieve a 20% fraction of patients assigned to validation: all patients (n=25) with multiple recuts of their biopsy tissue blocks, and patients (n=81) corresponding to a random selection of 119 slides stratified on ISUP grade (to be rescanned repeatedly over time for an AI temporal stability study). The STHLM3 subsets allocated into the internal validation set were selected based on being particularly valuable for the evaluation phase of the study, while the SUH subsets will be used as validation sets in upcoming follow-up studies involving the AI model developed here, hence cannot be assigned to the development set. The RUMC internal validation set includes the RUMC private test set in Kaggle (ie, PANDA RUMC internal validation set),^17^ supplemented with randomly sampled patients to achieve a total 20% fraction of patients assigned to tuning and validation.

External validation cohorts are fully external relative to the development set (no overlap with respect to patients, laboratory or scanner) or partly external (no overlap with respect to patients or laboratory, but digitisation performed using a scanner that is also present in the development set). Fully external validation set cohorts include Aichi Medical University (AMU), Aarhus University Hospital (AUH), KUH morphological subtypes (KUH-2), Mehiläinen Länsi-Pohja (MLP), Medical University of Lodz (MUL), Synlab Switzerland (SCH), Synlab Finland (SFI), Synlab France (SFR), Spear Prostate Biopsy 2020 (SPROB20), University Hospital Cologne (UKK), Hospital Wiener Neustadt (WNS). Partly external validation set cohorts include: Aquesta Uropathology morphological subtypes (AQ), partially scanned on a scanner present in the development set and partially scanned on an external scanner. The external nature of the validation set cohorts fulfils Requirements 4–6.

All data splits were performed on patient level, that is, all slides and resulting WSIs from a given patient were allocated to the same data partition in order to avoid information leakage between development and validation sets. If a patient was biopsied on several occasions, all biopsies were included and allocated together. Any samples lacking patient identifiers were assigned to development data to avoid the risk of slides from any patients ending up in both development and evaluation cohorts.

Subsets of the slides included in this study have been scanned multiple times. If the same slide had been rescanned multiple times on the same individual scanner (ie, the same physical device), we only kept the WSI with the latest scanning date, assuming the rescanning was due to, for example, initially poor focus or other scanning issues. Subsets of the STG, STHLM3 and MUL cohorts were rescanned with multiple different scanners (see table 1). To avoid biasing the evaluation towards these slides that appear in the dataset multiple times, we will only include one WSI per slide in the validation sets. For STHLM3, we will randomly select one WSI for each slide to be evaluated, and for MUL, we will use WSIs from the Grundium Ocus40 scanner, excluding those on the Philips UFS scanner. This ensures that the MUL cohort remains entirely external relative to the development data, considering that the STHLM3 cohort was partly digitised on the same Philips UFS instrument. The repeated scans will, however, be used during AI model development as an augmentation technique (except for the Grundium Ocus40 which is kept as an external scanner for validation), and for a separate cross-scanner reproducibility analysis (see section Statistical analyses).

### Statistical analyses

#### Overview of statistical analyses

1. Primary analysis: Diagnosis and Gleason scoring
  - Internal and external validation against the original cohort-specific reference standard
  - Subgroup analyses
    - Evaluate performance across different age groups.
    - Evaluate performance on systematic versus targeted biopsies.
    - Evaluate performance on non-treated patients versus patients treated for benign prostatic hyperplasia (BPH) prior to biopsy.
    - Evaluate performance on morphological subtypes.
    - Evaluate performance on cases requiring versus not requiring IHC staining.
    - Evaluate performance compared with the current state-of-the-art AI systems.
  - Sensitivity analyses
    - Cross-scanner consistency analyses.
    - Compare the AI system versus individual pathologist panel members.
    - Internal and external validation against uniform reference standard by the lead pathologist.
    - Blinded reassessment of slides with marked errors.
2. Secondary analysis: Cancer extent prediction
  - Internal and external validation against the original cohort-specific reference standards.
  - Subgroup analyses
    - Evaluate performance across different age groups.
    - Evaluate performance on systematic versus targeted biopsies.
    - Evaluate performance on non-treated patients vs patients treated for BPH prior to biopsy
  - Sensitivity analyses
    - Cross-scanner consistency analyses.
3. Secondary analysis: Cribriform cancer detection

- Internal and external validation against the original cohort-specific reference standards.
- Subgroup analyses
  - Evaluate performance across different age groups.
  - Evaluate performance on systematic versus targeted biopsies.
  - Evaluate performance on non-treated patients versus patients treated for BPH prior to biopsy.
- Sensitivity analyses

- Cross-scanner consistency analyses
- Compare the AI system versus individual pathologist panel members.
- Reassessment excluding borderline slides.

1. Secondary analysis: PNI detection
  - Internal and external validation against the original cohort-specific reference standards.
  - Subgroup analyses
    - Evaluate performance across different age groups.
    - Evaluate performance on systematic versus targeted biopsies.
    - Evaluate performance on non-treated patients versus patients treated for BPH prior to biopsy.
  - Sensitivity analyses
    - Cross-scanner consistency analyses
    - Compare the AI system versus individual pathologist panel members.
    - Reassessment excluding borderline slides.
2. Exploratory analyses
  - Evaluate visualisations of the AI output.
  - Evaluate the impact of tissue segmentation algorithms
  - Evaluate end-to-end versus transfer-learning-based models.
  - Evaluate the impact of physical colour calibration.

#### Details of statistical analyses

##### Primary analysis: diagnosis and Gleason scoring

We will quantify the concordance of the AI system’s cancer diagnosis (positive/negative), GS and ISUP grade with the reference standards in the tuning, internal validation and external validation cohorts using the metrics described below. The analysis will be conducted on slide level (AQ, AUH, KUH-1, KUH-2, MUL, RUMC, SFR, STHLM3, SUH, UKK, WNS), anatomical location level (MLP, SFI, SCH) and/or patient level (KUH-1, SCH, SFI, SFR, SPROB20) depending on the granularity of the available reference standards.

##### Cancer diagnosis

Sensitivity (true positive rate) and specificity (true negative rate) will be used to quantify the agreement of negative/positive diagnosis for prostate cancer with the reference standard. CIs for sensitivity and specificity will be computed using the non-parametric bootstrap over cases. We will additionally report the area under the receiver operating characteristics curve (AUROC) and confusion matrices.

##### Gleason score

Quadratically weighted Cohen’s kappa (QWK) will be used to quantify the agreement of Gleason scoring with the reference standard. In addition, we will also report linearly weighted Cohen’s kappa (LWK) and confusion matrices. To allow calculating weighted kappas, Gleason patterns (eg, 3+4) will be encoded into ordinal variables following earlier studies^43^,^45^ as follows: benign (0), 3+3 (1), 3+4 (2), 4+3 (3), 3+5 (4), 4+4 (5), 5+3 (6), 4+5 (7), 5+4 (8), 5+5 (9). CIs will be computed using the non-parametric bootstrap over cases.

##### ISUP grade

Quadratically weighted Cohen’s kappa (QWK) will be used to quantify the agreement of the ISUP grade with the reference standard. In addition, we will also report linearly weighted Cohen’s kappa (LWK) and confusion matrices. To allow calculating weighted kappas, ISUP grades will be treated as ordinal variables (0–5), with benign encoded as 0. CIs will be computed using the non-parametric bootstrap over cases.

### Secondary analysis: cancer extent prediction

We will quantify the concordance of the AI system’s prediction of linear cancer extent expressed in millimetres with the reference standards in those tuning, internal validation and external validation cohorts where a reference standard is available (AUH, KUH-1, STHLM3, SUH, STG, MLP, SCH, SFI, SFR). The concordance will be quantified using root mean squared error (RMSE). In addition, we will also report Pearson’s linear correlation coefficient and show scatter plots of predicted millimetre cancer length versus millimetre cancer length reported by the reference standard. The analysis will be conducted on slide level (AUH, KUH-1, STHLM3, SUH, STG, SFR), anatomical location level (MLP, SFI, SCH) and/or patient level (MLP, SCH, SFI, SFR) depending on the granularity of the available reference standards (see online supplemental appendix 2). CIs will be computed using the non-parametric bootstrap over cases.

### Secondary analysis: cribriform cancer detection

We will quantify the concordance of the AI system’s prediction of the presence of cribriform cancer with the reference standards in those internal and external validation cohorts where a reference standard is available (AMU, MUL, SCH, STHLM3, SUH). The tuning set has an insufficient number of cribriform samples for evaluation and will be included in the training. The concordance will be quantified using unweighted Cohen’s kappa. In addition, we will also report AUROC, sensitivity (true positive rate), specificity (true negative rate) and confusion matrices. Slides reported as borderline for cribriform cancer will be considered negative. The analysis will be conducted on slide level. CIs will be computed using the non-parametric bootstrap over cases.

The model will be developed and validated to identify cribriform growth pattern, irrespective of whether it occurs within acinar adenocarcinoma (ie, invasive cribriform) or intraductal carcinoma (IDC) (ie, non-invasive cribriform). The rationale for this is that these entities are often assessed and reported together for further prognostication and treatment planning. This practice is supported by the 2019 ISUP consensus, which recommended incorporating IDC into the Gleason grading when present alongside invasive carcinoma.^46^

### Secondary analysis: PNI detection

We will quantify the concordance of the AI system’s prediction of the presence of PNI with the reference standards in those internal and external validation cohorts where a reference standard is available (MUL, SCH, STHLM3 and SUH). The tuning set has an insufficient number of PNI samples for evaluation and will be included in the training. The concordance will be quantified using unweighted Cohen’s kappa. In addition, we will also report AUROC, sensitivity (true positive rate), specificity (true negative rate) and confusion matrices. Slides reported as borderline for PNI will be considered negative. The analysis will be conducted on slide level. CIs will be computed using the non-parametric bootstrap over cases.

### Subgroup analyses

#### Subgroup analysis A

We will measure the performance of the AI system in terms of the primary and secondary objectives across subgroups of patients divided by age. Analysis will be conducted on the cohorts where age information can be retrieved (online supplemental appendix 2) according to the age groups: <50, 50–59, 60–69 and ≥70.

#### Subgroup analysis B

We will measure the performance of the AI system in terms of the primary and secondary objectives across subgroups of patients divided by biopsy sampling technique (systematic vs targeted vs combined). The analysis will be conducted on the cohorts where biopsy sampling technique information can be retrieved.

#### Subgroup analysis C

We will measure the performance of the AI system in terms of the primary and secondary objectives across subgroups of patients who were treatment-naive or had received treatment for BPH (using, eg, 5-alpha reductase inhibitors) before the biopsy procedure. The analysis will be conducted on the cohorts where treatment information can be retrieved. Some (very few) individuals included in the patient cohorts may also have undergone prior prostate cancer treatment (eg, radiation therapy), but the number of cases is insufficient for a subgroup analysis.

#### Subgroup analysis D

We will measure the performance of the AI system in terms of the primary objective on subgroups of slides representing morphological subtypes of benign and malignant tissue that are usually hard for pathologists to diagnose. We evaluate the performance of the AI system in the STHLM3 morphological subtypes internal validation cohort, the KUH-2 external validation cohort and the AQ external and partly external validation cohorts. See table 4 for the distribution of morphological subtypes reported in each cohort. We will evaluate performance in terms of cancer diagnosis and, additionally, Gleason scoring, where applicable to the subtype.

#### Subgroup analysis E

We will measure the performance of the AI system in terms of the primary objective across subgroups of slides which required IHC staining for confirming the diagnosis and slides which the pathologists could assess without IHC. The analysis will be conducted on the cohorts where information on IHC can be retrieved (see table 5).

#### Subgroup analysis F

We will measure the performance of the AI system in terms of the primary objective in comparison to the state-of-the-art algorithms developed in the PANDA challenge.^17^ The analysis will be conducted on the subgroups of the KUH-1, RUMC and STHLM3 cohorts representing the internal and external validation sets of PANDA. For a fair comparison, we will apply the AI system on the WSIs provided to the challenge participants, which differ in terms of preprocessing and file format from the underlying original WSIs of the KUH-1 and STHLM3 cohorts, which are used in our primary analysis.

1. We evaluate the performance in the tuning cohort KUH-1 (ie, PANDA European external validation set) and compare the AI system with the PANDA challenge algorithms.
2. We evaluate the performance in the combined PANDA subset of the RUMC and STHLM3 internal validation cohorts (ie, PANDA internal validation set) and compare the AI system with the PANDA challenge algorithms.

### Sensitivity analyses

#### Sensitivity analysis A

We will evaluate the reproducibility of the AI system’s output in terms of the primary and secondary objectives on WSIs obtained from the same slides on multiple scanners. The analysis will be conducted on the STHLM3 tuning and internal validation cohorts and the MUL external validation cohort, which contain WSIs rescanned on different scanners (see table 1). In the STHLM3 cohort, a subset of slides (n=287) have been rescanned on five scanners: Aperio AT2 DX, Grundium Ocus40, Hamamatsu NanoZoomer 2.0-HT C9600-12, Hamamatsu NanoZoomer XR C12000-02 and Philips IntelliSite UFS. In the MUL cohort, a subset of slides (n=503) have been rescanned on two scanners: Grundium Ocus40 and Philips IntelliSite UFS. We will quantify the reproducibility of the AI predictions across scanners using QWK, and LWK and the percentage of slides with discordant predictions for each objective and each pair of scanners. We will additionally report confusion matrices.

#### Sensitivity analysis B

To put the discrepancies between the AI system and the reference standards in the context of interobserver variation between pathologists, we will quantify all-against-all pairwise agreements in panels consisting of pathologists and the AI system.

For the primary objective, the analysis will be conducted on subsets of the STHLM3 (ImageBase) and RUMC (PANDA Radboud) internal validation cohorts and on the full UKK and WNS external validation cohorts, which were assessed by a panel of pathologists and have per-pathologist grades available in addition to their consensus (see table 2). For the secondary objectives of cribriform cancer and PNI detection, the analysis will be conducted on subsets of the STHLM3 internal validation cohort, assessed by panels of pathologists (see table 3).

We will calculate the average pairwise agreement (QWK and LWK for the primary objective, unweighted Cohen’s kappa for the secondary objectives) for all the pathologists in the panels, including the AI system, and compare the average AI-pathologist agreement to the average pathologist-pathologist agreement. CIs will be computed using bootstrapping, as detailed in a previous study.^47^

#### Sensitivity analysis C

To assess the sensitivity of the results to different pathologists providing the cohort-specific reference standards and to isolate differences in observed AI performance due to varying reference standards from those due to imperfect generalisation to different labs and scanners, we will repeat the primary analysis using a consistent reference standard. We will measure the agreement between the AI system and the uniform reference standard set by the lead pathologist (LE) on subsets of the SUH and RUMC internal validation cohorts and the AUH, MLP, MUL, SCH, SFI, SFR and SPROB20 external validation cohorts. While the original reference standards were varyingly reported either on the level of slides, anatomical locations, or patients, LE’s reassessments are consistently reported on slide level. See table 2 for a summary of the reassessed subsets and reference standard protocol in online supplemental appendix 2 for details on the case selection for each cohort.

Furthermore, we will measure the agreement in ISUP grades (QWK and LWK) between the original reference standards and the lead pathologist on the reassessed subsets of each cohort. To facilitate this comparison for cohorts with original reference standards provided on anatomical location or patient level (whereas the grading by LE is on slide level), the location or patient level grading by LE will be obtained as the maximum ISUP grade over all slides belonging to a location or patient.

#### Sensitivity analysis D

We will perform a sensitivity analysis that involves a reassessment of slides where the AI system committed clinically significant errors by repeating the primary analysis against the updated reference standard. This analysis aims to evaluate what portion of clinically significant errors can be attributed to data quality issues, such as mistyped information in the reference standard tables, mixed-up slide identifiers or WSI scanning issues in cases where the original reference standard was set using a microscope. Significant errors are defined as cases where the AI model predicts a slide as benign, but the reference standard indicates ISUP grade ≥2, or conversely the AI predicts a slide as ISUP grade ≥2, but the reference standard indicates benign. These slides will be reassessed by the lead pathologist (LE) and/or other experienced uropathologists, blinded to the original reference standard and the AI output. If a slide cannot be assessed due to, for example, poor focus, it will be excluded. The evaluation will be conducted on the internal and external validation cohorts, on both the full cohorts after updating the reference standards, and on only the updated subsets. Additionally, during this analysis, pathologists will report whether any of the cases with clinically significant errors represent ductal adenocarcinoma (DAC). Despite being the second most common subtype of prostate cancer after acinar adenocarcinoma, DAC only accounts for 0.17% of prostate cancers^48^ and may, therefore, be challenging for AI to detect due to the limited amount of training data.

#### Sensitivity analysis E

We will perform a sensitivity analysis that involves the exclusion of samples reported by the pathologists as ‘borderline’ for cribriform cancer or PNI, followed by repeating the secondary analyses concerning these objectives. Conducting the analysis only on samples indicated as negative or positive will provide an estimate of the AI system’s performance in detecting cribriform cancer and PNI less affected by the uncertainty and subjectivity in the definition of these entities. We will additionally quantify the prevalence of borderline diagnoses among slides initially classified as false positives versus true negatives to quantify whether borderline cases are overrepresented among false positives. This would indicate that false positives mainly arise due to uncertainty of the reference standard.

### Exploratory analysis: evaluate visualisations of the AI output

We will output visualisations of the AI system’s predictions to highlight areas on each slide containing different Gleason patterns, cribriform cancer or PNI. The visualisations will be assessed qualitatively by the lead pathologist (LE) and/or other experienced uropathologists for concordance with their assessments. We may additionally quantify the rate of agreement between the AI system and the pathologists by collecting region annotations to serve as a reference standard, and by calculating the pixel-wise sensitivity, specificity, intersection over union or other suitable metrics.

### Exploratory analysis: evaluate the impact of tissue segmentation algorithms

Detecting tissue from the background to only apply the rest of the analysis on tissue pixels is a common preprocessing step for most computational pathology algorithms. While this task of tissue segmentation may seem trivial, many modern AI algorithms reach such low error rates in their main task that any errors in tissue detection can contribute to the overall model performance in a considerable way. In particular, missed tissue poses a risk of false negative diagnoses, if this leads to the exclusion of malignant tissue from the analysis. We will evaluate the effect of tissue segmentation on the overall performance of the AI system in terms of the primary and secondary objectives by comparing two different tissue segmentation algorithms. One of the algorithms represents classical image processing and relies on filtering and thresholding the image.^15^ The other algorithm is a trained deep learning-based segmentation model. We will apply both algorithms to perform the tissue segmentation during model training and validation and compare the results on the internal and external validation cohorts.

### Exploratory analysis: evaluate end-to-end versus transfer-learning-based models

Recently, so-called foundation models trained in a self-supervised manner on large and heterogeneous datasets have been proposed as generally applicable solutions to diverse tasks in computational pathology as an alternative to tissue type or task-specific models.^49^ We aim to compare our end-to-end trained prostate cancer-specific model to transfer-learning-based models relying on state-of-the-art foundation models for histopathology. We will apply a suitable foundation model as a feature extractor and train an additional classifier to adapt the model to the task of diagnosis and Gleason scoring of prostate biopsies. For this transfer learning step, we will use the same development cohorts as for the end-to-end trained model. We will then evaluate the model on the same internal and external validation cohorts as the end-to-end trained model for a direct comparison.

### Exploratory analysis: evaluate the impact of physical colour calibration

Variations in the reproduction of colour across different digital pathology scanners may pose a problem for AI, leading to inconsistent model outputs depending on the scanner used for slide digitisation. A physical calibrant in the form of a spectrophotometrically characterised slide has been proposed as a means for standardising the colour characteristics of WSIs acquired with different scanners.^50^ We will evaluate the impact of applying physical colour calibration on the performance of the AI model on those internal and external validation cohorts where the calibrant slide could be scanned on the same scanner as the prostate biopsies to allow calibration.

### Confounding factors

Statistical confounding, or spurious correlations, in the training and validation data of predictive models, may lead to ‘shortcut learning’ or so-called ‘Clever Hans predictors’,^51^ where overly optimistic performance on validation data is seen as the result of the model taking advantage of unintended correlations between some attributes of the data and the correct labels. Such biases are also common in digital pathology datasets.^20 22^ We have carefully considered the potential presence of such biases in our cohorts and taken the steps described below to mitigate the issue.

An important confounding factor is the scanner instruments used for digitising various subsets of our data cohorts. Patients in different cohorts and subsets of cohorts have been sampled in varying ways, leading to differences in the compositions of these groups in terms of GS and ISUP grade distribution. These correlations between specific clinical sites or scanner instruments and the target labels can create biases during training since the model could learn to associate the appearance of WSIs obtained from a specific site or with a specific scanner with a higher or lower likelihood of a particular diagnostic or grading outcome. If the same bias is present in validation data, this will lead to overly optimistic results. Conversely, if the bias present in training data is not present in the validation data, a model relying on these spurious correlations will perform poorly. The main approach we have taken to mitigate the risk of overly optimistic validation results is relying on fully external validation data. The external validation cohorts represent patients, clinical sites, laboratories and scanners not present in the training data. This minimises the risk of the same spurious correlations appearing in both training and external validation data. When it comes to discouraging the model from learning any spurious correlations between laboratories or scanners and the target labels, which could result in suboptimal performance in the absence of these correlations, we will apply a sampling scheme which removes the correlations between these variables during model training.

Another common confounding factor we have identified is markings on the slides. Pathologists often place pen marks on the glass slides to indicate cancerous regions. These can lead the AI model to directly associate the presence of markings with the presence of cancer, or indirectly to associate image quality artefacts such as poor focus caused by the pen marks with a higher likelihood of cancer being present. We have mitigated these issues by (1) applying tissue detection and masking of background pixels as an image preprocessing step, ensuring that pen markings adjacent to tissue will not be shown to the model, (2) washing and rescanning of slides where pen markings are placed on top of tissue or caused focusing issues or (3) excluding slides where neither of the first two options was possible. The first approach of background masking is applied to all the WSIs included in the study. The second approach of washing slides was applied to the development cohorts where we had control over the scanning process, namely STHLM3 and SUH. In the RUMC cohort, we excluded slides with pen marks on the tissue based on the findings of the participants in the PANDA challenge.

### Representative sampling

A key issue in the evaluation of diagnostic tests is how disease prevalence influences estimates of statistical measures used to assess the diagnostic performance of the tests. Prevalence is generally defined as the proportion of individuals in a population who have a particular disease at a given time. However, more specifically, the prevalence relates to the datasets used for evaluating a diagnostic test.

The positive predictive value (PPV; ie, the probability that individuals with a positive test result truly have the disease), negative predictive value (NPV; ie, the probability that individuals with a negative test result truly do not have the disease) and the Cohen’s kappa statistics are influenced by the disease prevalence in the datasets used for evaluating the performance of diagnostic tests. As prevalence increases, the PPV of a test also increases; and conversely, NPV decreases with increasing prevalence. This relationship means that in datasets where a disease (or disease subtype) is more common, the test’s ability to identify true positives increases and true negatives decreases. Similarly, the disease prevalence and case mix will impact estimates of Cohen’s kappa.

In contrast to PPV, NPV and Cohen’s kappa, sensitivity (also known as true positive rate, that is, the ability of a test to correctly identify patients with the disease) and specificity (also known as true negative rate, ie, the ability to correctly identify those without the disease) are not affected by changes in prevalence. These measures are intrinsic properties of the test and do not depend on how common the disease is in a population or dataset.

The sampling scheme or experimental design impacts the estimated prevalence in a study, thereby affecting the diagnostic performance statistics that are sensitive to prevalence. For example, in case–control studies, the prevalence is artificially set by the researcher. In datasets collected for the development of diagnostic AI systems (such as the one described in this protocol), it is common to upsample patients with a disease or disease subtype. If a consecutive case series were used for training an AI system to perform Gleason scoring, a very large set would be required in order to ensure a sufficiently large subsample of, for example, GS 9 and 10 samples for efficient training. Similarly, convenience sampling, where subjects are selected based on their availability rather than at random or according to a defined study design, can lead to a sample with a prevalence rate that does not match the general population. These types of experimental designs and sampling schemes can lead to assessments of PPV, NPV and Cohen’s kappa that do not reflect estimates that would be obtained in a consecutive case series in the general population.

The impact of prevalence on performance estimates underlines the importance of carefully considering the design of diagnostic studies. When prevalence is expected to differ, adjustments or different interpretations of PPV and NPV may be necessary to avoid misinformative conclusions. The data we use for training and evaluation of the AI system is a mixture of convenience samples (AMU, AQ, KUH-2, RUMC, SPROB20, STG) and data representing consecutive clinical cases or another well-defined and controlled sampling scheme (AUH, KUH-1, MLP, MUL, SCH, SFI, SFR, STHLM3, SUH, UKK, WNS). For the datasets with a known sampling scheme and experimental design, we can use prior probability shift corrections to achieve estimates of PPV, NPV and Cohen’s kappa on a well-defined base population.^52 53^

### Power

We have not performed formal power (or sample size) calculations. The reason for this is as follows:

- The central objective of this study is to calculate point estimates of performance (using statistical measures as described above) and their CIs, rather than emphasising power to detect a specific effect size (which is more relevant when comparing interventions or diagnoses).
- This is a retrospective evaluation of AI for prostate pathology. This means that the sample size is fixed based on the datasets at hand.

## Ethics and dissemination

The study is conducted in agreement with the Declaration of Helsinki. The data were retrieved in one or more rounds at each of the participating international sites between 1 May 2012 and 1 May 2024. All data were deidentified at each site and provided to Karolinska Institutet in anonymised format. The centralised collection of patient samples from the international sites to Karolinska Institutet was approved by the Swedish Ethical Review Authority (permit 2019-05220). The following local approvals were provided to cover the data collection at each site: AMU (permit 2023-074 for the AMU cohort), Aquesta Pathology Ethics Committee (permit 2023-001: 462 351 for the AQ cohort), Stockholm regional ethics committee (permits 2012/572-31/1, 2012/438-31/3, and 2018/845-32 for the KS, STG and STHLM3 cohorts), the Bioethics Committee at the Medical University of Lodz (permit RNN/295/19/KE for the MUL cohort), and the Regional Committee for Medical and Health Research Ethics (REC) in Western Norway (permits REC/Vest 80924, REK 2017/71 for the SUH cohort). For the AUH, MLP, SCH, SFI and SFR cohorts, ethical approval was waived by the respective local institutional review boards due to the retrospective usage of fully deidentified prostate specimens, and the data collection under the waiver was approved by the Swedish Ethical Review Authority (permit 2019-05220). The RUMC, SPROB20, UKK and WNS cohorts represent fully deidentified open data available in online repositories (see online supplemental appendix 2 for details). Written informed consent was provided by the participants in the STHLM3 dataset. For the other datasets, informed consent was waived by the institutional review boards due to the usage of deidentified prostate specimens in a retrospective setting, and the data collection under the waiver was approved by the Swedish Ethical Review Authority (permit 2019-05220). The study results will be submitted for publication in an open-access format, regardless of whether the findings are positive, negative or inconclusive in relation to the study hypothesis.

### Study status

The key time points for a retrospective AI development and validation study are: (1) establishment of the prespecified statistical analysis to be conducted on validation data, (2) locking of the AI model design, (3) unlocking of the validation data to evaluate the final model’s diagnostic performance and, potentially, (4) repeating evaluation on additional validation datasets while the model design remains locked. Respecting this timeline is crucial to ensure there is no information leakage from the validation data to influence the AI model design. Development data, on the other hand, can be freely accessed at any point, allowing optimisation of the AI model design prior to the validation phase of the study. The study status on this timeline is as follows:

1. 7 July 2024: The protocol was made publicly available as a preprint on medRxiv (https://www.medrxiv.org/content/10.1101/2024.07.04.24309948v1) to prespecify the analysis plan, presented here without modifications to its contents.
2. 16 August 2024: The AI model design was locked. No further changes to the model are allowed.
3. 2025: Final evaluation of the AI model will be conducted according to the prespecified analysis plan on the validation data and results published in a peer-reviewed journal.
4. TBA: The study has no specific completion date, as the study design will allow extension of the analysis with additional patient cohorts indefinitely, repeating the same analysis steps, as long as the AI model design will remain locked. We are currently collecting additional data cohorts to cover a wider representation of patient demographics (collection ongoing in Canada, Iraq, Italy and Jordan) and will reapply the locked AI model and analysis plan to validate AI performance on these cohorts. The results from these cohorts will be published separately in peer-reviewed journals when available.

## Supplementary material

10.1136/bmjopen-2024-097591 online supplemental file 1

10.1136/bmjopen-2024-097591 online supplemental file 2

## References

[1] Filho AM, Laversanne M, Ferlay J (2025). The GLOBOCAN 2022 cancer estimates: Data sources, methods, and a snapshot of the cancer burden worldwide. Int J Cancer.

[2] Loeb S, Vellekoop A, Ahmed HU (2013). Systematic review of complications of prostate biopsy. Eur Urol.

[3] James ND, Tannock I, N’Dow J (2024). The Lancet Commission on prostate cancer: planning for the surge in cases. Lancet.

[4] Gleason DF (1992). Histologic grading of prostate cancer: a perspective. Hum Pathol.

[5] Epstein JI, Allsbrook WC, Amin MB (2005). ISUP grading committee.

[6] Melia J, Moseley R, Ball RY (2006). A UK-based investigation of inter- and intra-observer reproducibility of Gleason grading of prostatic biopsies. Histopathology.

[7] Ozkan TA, Eruyar AT, Cebeci OO (2016). Interobserver variability in Gleason histological grading of prostate cancer. Scand J Urol.

[8] Egevad L, Ahmad AS, Algaba F (2013). Standardization of Gleason grading among 337 European pathologists. Histopathology.

[9] Epstein JI, Egevad L, Amin MB (2016). The 2014 International Society of Urological Pathology (ISUP) Consensus Conference on Gleason Grading of Prostatic Carcinoma: Definition of Grading Patterns and Proposal for a New Grading System. Am J Surg Pathol.

[10] International Agency for Research on Cancer (2022). WHO classification of tumours of the urinary system and male genital organs.

[11] Egevad L, Delahunt B, Iczkowski KA (2023). Interobserver reproducibility of cribriform cancer in prostate needle biopsies and validation of International Society of Urological Pathology criteria. Histopathology.

[12] Egevad L, Delahunt B, Samaratunga H (2021). Interobserver reproducibility of perineural invasion of prostatic adenocarcinoma in needle biopsies. Virchows Arch.

[13] Pantanowitz L, Sharma A, Carter AB (2018). Twenty Years of Digital Pathology: An Overview of the Road Travelled, What is on the Horizon, and the Emergence of Vendor-Neutral Archives. J Pathol Inform.

[14] Campanella G, Hanna MG, Geneslaw L (2019). Clinical-grade computational pathology using weakly supervised deep learning on whole slide images. Nat Med.

[15] Ström P, Kartasalo K, Olsson H (2020). Artificial intelligence for diagnosis and grading of prostate cancer in biopsies: a population-based, diagnostic study. Lancet Oncol.

[16] Bulten W, Pinckaers H, van Boven H (2020). Automated deep-learning system for Gleason grading of prostate cancer using biopsies: a diagnostic study. Lancet Oncol.

[17] Bulten W, Kartasalo K, Chen P-HC (2022). Artificial intelligence for diagnosis and Gleason grading of prostate cancer: the PANDA challenge. Nat Med.

[18] Ji X, Salmon R, Mulliqi N (2025). Physical Color Calibration of Digital Pathology Scanners for Robust Artificial Intelligence–Assisted Cancer Diagnosis. Mod Pathol.

[19] Swiderska-Chadaj Z, de Bel T, Blanchet L (2020). Impact of rescanning and normalization on convolutional neural network performance in multi-center, whole-slide classification of prostate cancer. Sci Rep.

[20] Schmitt M, Maron RC, Hekler A (2021). Hidden Variables in Deep Learning Digital Pathology and Their Potential to Cause Batch Effects: Prediction Model Study. J Med Internet Res.

[21] Duenweg SR, Bobholz SA, Lowman AK (2023). Whole slide imaging (WSI) scanner differences influence optical and computed properties of digitized prostate cancer histology. J Pathol Inform.

[22] Howard FM, Dolezal J, Kochanny S (2021). The impact of site-specific digital histology signatures on deep learning model accuracy and bias. Nat Commun.

[23] Nagendran M, Chen Y, Lovejoy CA (2020). Artificial intelligence versus clinicians: systematic review of design, reporting standards, and claims of deep learning studies. BMJ.

[24] McGenity C, Bossuyt P, Treanor D (2022). Reporting of Artificial Intelligence Diagnostic Accuracy Studies in Pathology Abstracts: Compliance with STARD for Abstracts Guidelines. J Pathol Inform.

[25] Cruz Rivera S, Liu X, Chan A-W (2020). Guidelines for clinical trial protocols for interventions involving artificial intelligence: the SPIRIT-AI extension. Lancet Digit Health.

[26] Liu X, Cruz Rivera S, Moher D (2020). Reporting guidelines for clinical trial reports for interventions involving artificial intelligence: the CONSORT-AI extension. Lancet Digit Health.

[27] Vasey B, Nagendran M, Campbell B (2022). Reporting guideline for the early-stage clinical evaluation of decision support systems driven by artificial intelligence: DECIDE-AI. Nat Med.

[28] Collins GS, Dhiman P, Andaur Navarro CL (2021). Protocol for development of a reporting guideline (TRIPOD-AI) and risk of bias tool (PROBAST-AI) for diagnostic and prognostic prediction model studies based on artificial intelligence. BMJ Open.

[29] Collins GS, Moons KGM, Dhiman P (2024). TRIPOD+AI statement: updated guidance for reporting clinical prediction models that use regression or machine learning methods. BMJ.

[30] Sounderajah V, Ashrafian H, Golub RM (2021). Developing a reporting guideline for artificial intelligence-centred diagnostic test accuracy studies: the STARD-AI protocol. BMJ Open.

[31] Kleppe A, Skrede O-J, De Raedt S (2021). Designing deep learning studies in cancer diagnostics. Nat Rev Cancer.

[32] Mongan J, Moy L, Kahn CE (2020). Checklist for Artificial Intelligence in Medical Imaging (CLAIM): A Guide for Authors and Reviewers. *Radiol Artif Intell*.

[33] Tejani AS, Klontzas ME, Gatti AA (2023). Updating the Checklist for Artificial Intelligence in Medical Imaging (CLAIM) for reporting AI research. Nat Mach Intell.

[34] Park SH, Han K (2018). Methodologic Guide for Evaluating Clinical Performance and Effect of Artificial Intelligence Technology for Medical Diagnosis and Prediction. Radiology.

[35] Kweldam CF, Nieboer D, Algaba F (2016). Gleason grade 4 prostate adenocarcinoma patterns: an interobserver agreement study among genitourinary pathologists. Histopathology.

[36] Egevad L, Cheville J, Evans AJ (2017). Pathology Imagebase-a reference image database for standardization of pathology. Histopathology.

[37] Goode A, Gilbert B, Harkes J (2013). OpenSlide: A vendor-neutral software foundation for digital pathology. J Pathol Inform.

[38] Mulliqi N, Kartasalo K, Olsson H (2021). OpenPhi: an interface to access Philips iSyntax whole slide images for computational pathology. Bioinformatics.

[39] Varoquaux G, Cheplygina V (2022). Machine learning for medical imaging: methodological failures and recommendations for the future. *NPJ Digit Med*.

[40] Willemink MJ, Koszek WA, Hardell C (2020). Preparing Medical Imaging Data for Machine Learning. *Radiology*.

[41] Kartasalo K, Ström P, Ruusuvuori P (2022). Detection of perineural invasion in prostate needle biopsies with deep neural networks. Virchows Arch.

[42] Olsson H, Kartasalo K, Mulliqi N (2022). Estimating diagnostic uncertainty in artificial intelligence assisted pathology using conformal prediction. Nat Commun.

[43] Jung M, Jin M-S, Kim C (2022). Artificial intelligence system shows performance at the level of uropathologists for the detection and grading of prostate cancer in core needle biopsy: an independent external validation study. Mod Pathol.

[44] Egevad L, Micoli C, Delahunt B (2024). Prognosis of Gleason score 8 prostatic adenocarcinoma in needle biopsies: a nationwide population-based study. Virchows Arch.

[45] Egevad L, Micoli C, Samaratunga H (2024). Prognosis of Gleason Score 9-10 Prostatic Adenocarcinoma in Needle Biopsies: A Nationwide Population-based Study. Eur Urol Oncol.

[46] van Leenders GJLH, van der Kwast TH, Grignon DJ (2020). The 2019 International Society of Urological Pathology (ISUP) Consensus Conference on Grading of Prostatic Carcinoma. Am J Surg Pathol.

[47] Egevad L, Delahunt B, Berney DM (2018). Utility of Pathology Imagebase for standardisation of prostate cancer grading. Histopathology.

[48] Ranasinha N, Omer A, Philippou Y (2021). Ductal adenocarcinoma of the prostate: A systematic review and meta-analysis of incidence, presentation, prognosis, and management. *BJUI Compass*.

[49] Chen RJ, Ding T, Lu MY (2024). Towards a general-purpose foundation model for computational pathology. Nat Med.

[50] Clarke EL, Revie C, Brettle D (2018). D evelopment of a novel tissue‐mimicking color calibration slide for digital microscopy. Color Research & Application.

[51] Lapuschkin S, Wäldchen S, Binder A (2019). Unmasking Clever Hans predictors and assessing what machines really learn. Nat Commun.

[52] Schölkopf B, Janzing D, Peters J (2012). On causal and anticausal learning.

[53] Heiser TJT, Allikivi M-L, Kull M (2020). Shift happens: adjusting classifiers. Machine learning and knowledge discovery in databases.

