# Supplementary material for: Development and retrospective validation of an artificial intelligence system for diagnostic assessment of prostate biopsies: study protocol
Source: BMJ Open. 2025 Jul 7;15(7):e097591. doi: 10.1136/bmjopen-2024-097591 (PMC12258300; doi:10.1136/bmjopen-2024-097591)
Supplement: online supplemental file 1 [file bmjopen-15-7-s001.pdf]

Study Protocol: Development and Retrospective Validation of an Artificial Intelligence System for Diagnostic Assessment of Prostate Biopsies

Supplementary Appendix 1

Nita Mulliqi[1], Anders Blilie[2,3], Xiaoyi Ji[1], Kelvin Szolnoky[1], Henrik Olsson[1], Matteo Titus[1], Geraldine Martinez Gonzalez[1], Sol Erika Boman[1,4], Masi Valkonen[5], Einar Gudlaugsson[2], Svein R. Kjosavik[3,6], José Asenjo[7], Marcello Gambacorta[8], Paolo Libretti[8], Marcin Braun[9], Radzislaw Kordek[9], Roman Łowicki[10], Kristina Hotakainen[11,12], Päivi Väre[13], Bodil Ginnerup Pedersen[14,15], Karina Dalsgaard Sørensen[15,16], Benedicte Parm Ulhøi[17], Mattias Rantalainen[1], Pekka Ruusuvuori[4,18], Brett Delahunt[19], Hemamali Samaratunga[20], Toyonori Tsuzuki[21], Emilius A.M. Janssen[2,22], Lars Egevad[23], Kimmo Kartasalo[24], Martin Eklund[1]

1. Department of Medical Epidemiology and Biostatistics, Karolinska Institutet, Stockholm, Sweden
2. Department of Pathology, Stavanger University Hospital, Stavanger, Norway
3. Faculty of Health Sciences, University of Stavanger, Stavanger, Norway
4. Department of Molecular Medicine and Surgery, Karolinska Institutet, Stockholm, Sweden
5. Institute of Biomedicine, University of Turku, Turku, Finland
6. The General Practice and Care Coordination Research Group, Stavanger University Hospital, Norway
7. Department of Pathology, Synlab, Madrid, Spain
8. Department of Pathology, Synlab, Brescia, Italy
9. Department of Pathology, Chair of Oncology, Medical University of Lodz, Lodz, Poland
10. 1st Department of Urology, Medical University of Lodz, Lodz, Poland
11. Department of Clinical Chemistry, University of Helsinki, Helsinki, Finland
12. Laboratory Services, Mehiläinen Oy, Helsinki, Finland
13. Mehiläinen Länsi-Pohja Hospital, Kemi, Finland
14. Department of Radiology, Aarhus University Hospital, Aarhus, Denmark
15. Department of Clinical Medicine, Aarhus University, Aarhus, Denmark
16. Department of Molecular Medicine, Aarhus University Hospital, Aarhus, Denmark
17. Department of Pathology, Aarhus University Hospital, Aarhus, Denmark
18. Faculty of Medicine and Health Technology, Tampere University, Tampere, Finland
19. Department of Pathology and Molecular Medicine, Wellington School of Medicine and Health Sciences, University of Otago, Wellington, New Zealand
20. Aquesta Uropathology and University of Queensland, QLD, Brisbane, Australia
21. Department of Surgical Pathology, School of Medicine, Aichi Medical University, Nagoya, Japan
22. Faculty of Science and Technology, University of Stavanger, Stavanger, Norway
23. Department of Oncology and Pathology, Karolinska Institutet, Stockholm, Sweden
24. Department of Medical Epidemiology and Biostatistics, SciLifeLab, Karolinska Institutet, Stockholm, Sweden

1. ARTIFICIAL INTELLIGENCE SYSTEM CONSIDERATIONS **2**
2. DATA QUALITY AND LABEL NOISE **5**
3. VERIFICATION OF DATASET COHERENCE **6**
4. REFERENCES **8**

1. ARTIFICIAL INTELLIGENCE SYSTEM CONSIDERATIONS

To accelerate the development process by reducing runtime for early model designs and to simplify troubleshooting, we will initially only use one of the development cohorts for model training and gradually introduce the other development cohorts one by one. This approach to model development allows:

- Effective troubleshooting: systematic experiments facilitate easier debugging and identification of error root causes.
- Traceability and accountability: transparency and traceability of how the model evolved during development, and accountability in cases of improvements or issues.
- Isolation of changes: the impact of each modification is assessed independently without the confounding effects of simultaneous changes (e.g. changing multiple hyperparameters at once).
- Optimal model tuning: controlled and sequential modifications allow for optimal tuning of the model and achieving the best possible model performance.

The artificial intelligence (AI) system development will follow a comprehensive experimental pipeline driven by specific requirements and algorithmic considerations determining the model design and hyperparameter choices. These considerations encompass various aspects of AI design such as the granularity of data annotations, memory constraints, class imbalance, loss functions, training optimisation, and visualisation outputs. Below we list the design choices, which will be systematically evaluated during model development:

- Data preprocessing:
    - Explore different image preprocessing parameters (e.g. hue, saturation, morphological operations like closing size etc.) and convolutional neural network (CNN) algorithms to segment the tissue from the background.
    - Determine the optimal parameters for extracting tiles from whole slide images (WSIs), i.e. tile size, pixel size (2.0 µm, 1.5 µm, 1.0 µm, 0.5 µm), the extent of overlap between adjacent tiles and the minimum amount of tissue required in each tile. These configurations should balance computational efficiency, memory consumption and the level of granularity needed for accurate predictions.
    - Investigate the effect of the chosen tiling parameters, separately, for model training and prediction, considering the different memory consumption constraints during each phase. Investigate if combined tiling parameters affect the performance.
- Data storage:

    - Explore different disk-friendly formats for storing tiles, due to the large number of tiles produced per WSI.
- Bias and data quality:
    - Address biases related to the data that could potentially mislead the model during training, e.g. confounding factors on the slides (pen mark annotations from pathologists) or unequal class distributions between slides scanned with different scanning equipment.
- Data augmentation:
    - Apply diverse augmentations to increase robustness to variations in the input data and enhance the model's ability to generalise to unseen samples.
    - Investigate different augmentation techniques such as affine transformations (rotations, vertical or horizontal flipping), noise-simulating augmentations (Gaussian noise, ISO noise, image compression), colour and blur augmentations, and stain augmentations [1,2].
    - Investigate the effect of test time augmentation (TTA) techniques only, or in addition to the data augmentation during training.
    - Investigate if data augmentations should be conducted on the tile level or the WSI level.
    - Explore how to best utilise different WSIs of the same biopsy slide during model training e.g. sample a random WSI per slide or use all WSIs on each training epoch.
    - Explore the effect of using physical scanner calibration as a data augmentation or normalisation technique [3].
- Training data:
    - Sequentially incorporate additional development cohorts as training data.
    - Evaluate the effect of adding more training data for generalising better across different labs and pathology scanners.
- Model design:
    - Investigate learning strategies for handling bags of data where annotations are available at the WSI level rather than the tile level, e.g. self-supervised learning or weakly-supervised learning.
    - Explore design ideas from the top-performing PANDA teams [4].
    - Investigate the effect of encoder architectures and the number of parameters e.g. ResNet18, ResNet34, ResNet50, ResNet101 [5], EfficientNetV2-S, EfficientNetV2-M and EfficientNetV2-L [6] or foundation models [7,8].
    - Investigate aggregation mechanisms for obtaining both WSI-level predictions and tile-level visualisations (e.g. attention heatmaps).
    - Explore multitask learning to predict more than one outcome with a single model, e.g., Gleason score (GS) and cancer length.
- Model hyperparameters:

    - Experiment with different conventional learning rates (LR) (e.g. 0.0001, >0.0001, <0.0001) or cyclical LR for balancing learning speed and training stability.
    - Experiment with different optimisers (e.g. Adam/AdamW or stochastic gradient descent, SGD) for better convergence [9].
    - Experiment with different batch sizes: different number of WSIs and number of tiles per WSI.
- Loss functions:
    - Experiment with different loss functions: regression loss (mean squared error, MSE or mean absolute error, MAE), ordinal loss, cross-entropy loss, Coral loss [10] etc.
- Memory optimisation and computational efficiency:
    - Experiment on available hardware on high-performance computing clusters to support larger effective batch sizes without exceeding memory limits.
    - Explore the effect of mixed precision during training and prediction for reduced memory consumption and increased throughput [11].
    - Explore the effect of checkpointing gradients [12] for reduced memory footprint at the cost of additional computation time during training.
    - Explore the effect of accumulating gradients over multiple mini-batches before performing the optimiser to obtain larger effective batch sizes without requiring additional memory.
    - Explore employing PyTorch Distributed Data-Parallel (DDP) for multi-node, multi-graphical processing unit (GPU) training to speed up training [13].
- Model output:
    - Investigate different model heads for predicting Gleason patterns, GS, or the International Society of Urological Pathology (ISUP) grade.
    - In the case of predicting GS or patterns using a regression loss function, investigate how to best map those into ordinal variables [14–16].
- Early stopping criteria:
    - Experiment with monitoring the validation loss or other metrics (e.g. Cohen's linearly or quadratically weighted kappa, LWK/QWK) and halting training when the metric reaches a plateau.
    - Monitor performance and optimise early stopping parameters, including the minimum and the maximum number of epochs and the patience threshold for terminating training.
- Handle class imbalance:
    - Experiment with a weighted loss function to penalise misclassifications of the minority class more heavily than the majority class.
    - Experiment with sampling to balance class frequencies for each training epoch.
- Model ensembles:
    - Experiment with model ensembles across and within cross-validation folds.

- Experiment with using different random number generator seeds for each cross-validation fold.
- Define an optimal number of within-fold and across-fold models for the ensemble.
- Experiment with varying methods of ensembling e.g. hard-voting and soft-voting.

2. DATA QUALITY AND LABEL NOISE

Collecting and pseudonymising or anonymising clinical and pathology data and associating these records with the correct WSIs requires a number of steps, each introducing potential sources for error. Our data collection, management and verification process generally followed these steps:

Retrieval and digitisation of clinical/pathology data: Depending on the data cohort, the clinical and pathology data were extracted from existing databases/registries (STHLM3) in tabular form, provided in tabular form by the data providing sites (AMU, AQ, AUH, MLP, MUL, RUMC, SPROB20, SUH, UKK, WNS) or tabulated manually in-house from pathology reports scanned into PDF files (KUH-1, KUH-2, SCH, SFI, SFR, STG). The manual tabulation in-house involved human translation of the reports from Finnish (SFI), French (SCH, SFR) and Swedish (KUH-1, KUH-2, STG) by trained non-experts fluent in the respective languages. Patient identifiers were pseudonymised during the data extraction or tabulation process by each data provider.

Retrieval and digitisation of slides: Slides were retrieved from the respective archives at each site and scanned with the instruments tabulated in Table B in Supplementary Appendix 2. Each slide had a label with an identifier and depending on the scanning site, the identifiers were stored either in the form of macro/label images as part of the WSI metadata, automatically detected from QR codes and stored as WSI metadata, or manually typed in by the scanner operator when naming the resulting WSI files.

Linking slides to clinical/pathology data: Depending on the manner in which slide identifiers were stored for each WSI, the linking step involved one of the following approaches. For WSIs, where the identifier was manually typed into the filename, customised scripts were written in Python for each data cohort to parse the filename strings. This involved comparing the parsed identifiers to those present in the clinical/pathology data, and iterative refinements to rectify issues such as missing or additional zeros, missing or additional whitespace or other delimiters, and discrepancies with the representation of characters not belonging to the Basic Latin (standard ASCII) set, e.g. Ä or Ö. For WSIs, where the identifier was stored in the form of WSI metadata, we used an in-house developed optical character recognition (OCR) system to extract identifiers in a semi-automated manner from the QR-code based metadata items and the macro/label images embedded in the WSIs. The system first extracted the QR-code based identifier, if available, or

performed OCR using the *pytesseract* (version 0.3.2) implementation of the Tesseract OCR engine [17]. The system featured a simple user interface, which presented the automatically detected identifier pre-filled into a text box, alongside the macro/label image of the slide. The human operator then had the option of accepting the proposed identifier or correcting it manually based on the label image. All identifiers were assessed by trained non-experts using this semi-automated approach.

Relabeling: Slides and patients were initially labelled independently by each data provider using pseudonymised identifiers. This poses a risk that the same identifier (e.g. Patient_01) is used by multiple data providers, which would cause ambiguous matches in the final combined dataset. In order to minimise this risk and to obtain unique identifiers for each WSI, each slide and each patient, we calculated unique MD5 hashes based on the variables below. This step additionally provided another round of pseudonymisation to minimise the risk of any non-pseudonymised identifiers being accidentally used by the data providing sites.

- WSI ID: Filename + scanner serial number + scanning time stamp
- Slide ID: Cohort name + original slide ID
- Patient ID: Cohort name + original patient ID

Verification: The final dataset covering all the data cohorts is managed internally as a CSV spreadsheet, generated and maintained using scripts written in Python relying on *pandas* [18]. Upon generation and any modifications, the dataset undergoes comprehensive unit testing to ensure correctness, implemented in Python using the *unittest* framework. A version history of the dataset is retained to allow tracing back errors. In summary, the tests used for verification cover the following aspects. The uniqueness and unambiguity of matches based on the identifiers described above are verified. Patient-level variables are tested for consistency across all slides and WSIs from the same patient, and slide level variables are checked for consistency across multiple WSIs representing the same slide. We verify that all variables have valid values, with specific tests for categorical, quantitative, and Boolean variables and test for logical mismatches between variables (e.g. a slide negative for cancer cannot be positive for PNI). We ensure there is no overlap between patients in different development vs. validation splits or between cross-validation folds in the development data. Please refer to the list below for an extensive explanation of all the tests.

3. VERIFICATION OF DATASET COHERENCE

The datasets used in this study undergo rigorous testing to maintain data integrity and accuracy. Below is a list of all tests performed programmatically whenever the dataset is updated. Here, “main database” refers to a spreadsheet containing all the data, where each row represents a single whole slide image (WSI) along with its associated pathology information (e.g. Gleason score (GS) or International Society of Urological Pathology (ISUP) grade etc.), scanning and

digitisation details and data partition. Each WSI, slide (i.e. one glass slide containing one or more tissue cores), “location” (i.e. a set of slides treated as a single diagnostic unit and graded together, sometimes called “part” or “block” but not to be confused with a paraffin block), biopsy (i.e. a single operation during which multiple tissue cores are obtained from the prostate) and patient in the database is represented by a unique identifier (ID), and the data are partitioned into development, tuning, validation or exclusion.

- Consistency in data partitions:
    - All patients assigned to the data partitions ("development", "tuning", "validation", "exclusion") must be present in the main database.
    - All WSIs in the main database should be assigned to a data partition ("development", "tuning", "validation", "exclusion").
    - All WSIs in the "development" partition and none of the WSIs in the other partitions should be assigned to a cross-validation fold.
    - Patients must not overlap between data partitions (i.e. a patient can belong to one partition and one partition only).
    - Partition assignments must be consistent for all WSIs and slides of a patient.
    - Verify that all the patients belonging to special subsets intended for internal validation are assigned to “validation” i.e. ImageBase, perineural invasion (PNI) validation set, PANDA private test sets, morphological subtypes, Stavanger University Hospital (SUH) repeated scanning set, and SUH patients with re-cuts.

- Consistency for variables across patients, biopsies, slides and WSIs:
    - Patient level variables (e.g. age) and slide level variables (e.g. Gleason score) must be consistent across all WSIs representing the same patient or the same slide, respectively. (The requirement for consistent Gleason scoring is relaxed in the case of slides re-graded for PANDA.)
    - Biopsy level variables must be consistent across the WSIs representing the same biopsy procedure (e.g. age at the time of the biopsy or prostate-specific antigen, PSA etc.) since some cohorts include patients who have undergone multiple biopsies at different times.
    - Location level variables must be consistent across the WSIs representing the same “location”. Location IDs must match slide IDs for cohorts with slide level reporting.

- Validation of variable values:
    - Verify that all variables in the database have at least one non-empty entry.
    - Verify that the following variables are non-empty for all WSIs: WSI ID (identifier unique to each dataset entry), ID (unique identifier for each slide), Patient ID

(unique identifier for each patient), Pathology ID (identifier unique to a single biopsy procedure), path and filename of the WSI, and data cohort.

- There must not be any duplicated WSI IDs.
- There must not be any duplicated WSI paths or filenames.
- GS and ISUP grade per slide, location or patient must have valid values i.e. ("0 + 0", "3 + 3", "3 + 4", "4 + 3", "4 + 4", "3 + 5", "5 + 3", "4 + 5", "5 + 4", "5 + 5") and ("0", "1", "2", "3", "4", "5"), respectively, or be empty.
- If both the GS and ISUP grade per slide/location/patient are present, they must follow the ISUP grade definition (e.g. GS 3 + 3 must be ISUP 1).
- At least one grading variable (GS, GS patient, ISUP, ISUP patient) must be populated, except for the morphological subtype cohorts.
- Quantitative variables (e.g. cancer length, biopsy length) must be non-negative or empty.
- Percentual variables (e.g. cancer percentage) must be 0-100 or empty.
- Variables for cribriform cancer and PNI must be True, False, Borderline or empty.
- Boolean variables must be True, False, or empty.

4. REFERENCES

1. Faryna K, van der Laak J, Litjens G. Automatic data augmentation to improve generalization of deep learning in H&E stained histopathology. Comput Biol Med. 2024;170: 108018.

2. Faryna K, van der Laak J, Litjens G. Tailoring automated data augmentation to H&E-stained histopathology. In: Heinrich M, Dou Q, de Bruijne M, Lellmann J, Schläfer A, Ernst F, editors. Proceedings of the Fourth Conference on Medical Imaging with Deep Learning. PMLR; 07--09 Jul 2021. pp. 168–178.

3. Ji X, Salmon R, Mulliqi N, Khan U, Wang Y, Blilie A, et al. Physical Color Calibration of Digital Pathology Scanners for Robust Artificial Intelligence Assisted Cancer Diagnosis. arXiv [q-bio.QM]. 2023. Available: http://arxiv.org/abs/2307.05519

4. Bulten W, Kartasalo K, Chen P-HC, Ström P, Pinckaers H, Nagpal K, et al. Artificial intelligence for diagnosis and Gleason grading of prostate cancer: the PANDA challenge. Nat Med. 2022;28: 154–163.

5. He K, Zhang X, Ren S, Sun J. Deep residual learning for image recognition. Proceedings of the IEEE conference on computer vision and pattern recognition. 2016. pp. 770–778.

6. Tan M, Le Q. EfficientNetV2: Smaller Models and Faster Training. In: Meila M, Zhang T, editors. Proceedings of the 38th International Conference on Machine Learning. PMLR; 18--24 Jul 2021. pp. 10096–10106.

7. Chen RJ, Ding T, Lu MY, Williamson DFK, Jaume G, Song AH, et al. Towards a

general-purpose foundation model for computational pathology. Nat Med. 2024;30: 850–862.

8. Xu H, Usuyama N, Bagga J, Zhang S, Rao R, Naumann T, et al. A whole-slide foundation model for digital pathology from real-world data. Nature. 2024. doi:10.1038/s41586-024-07441-w

9. Kingma DP, Ba J. Adam: A Method for Stochastic Optimization. arXiv [cs.LG]. 2014. Available: http://arxiv.org/abs/1412.6980

10. Sun B, Saenko K. Deep CORAL: Correlation Alignment for Deep Domain Adaptation. Computer Vision – ECCV 2016 Workshops. Springer International Publishing; 2016. pp. 443–450.

11. Micikevicius P, Narang S, Alben J, Diamos G, Elsen E, Garcia D, et al. Mixed Precision Training. arXiv [cs.AI]. 2017. Available: http://arxiv.org/abs/1710.03740

12. Rumelhart DE, Hinton GE, Williams RJ. Learning representations by back-propagating errors. Nature. 1986;323: 533–536.

13. Paszke A, Gross S, Chintala S, Chanan G, Yang E, DeVito Z, et al. Automatic differentiation in PyTorch. 2017. Available: https://openreview.net/pdf?id=BJJsrmfCZ

14. Jung M, Jin M-S, Kim C, Lee C, Nikas IP, Park JH, et al. Artificial intelligence system shows performance at the level of uropathologists for the detection and grading of prostate cancer in core needle biopsy: an independent external validation study. Mod Pathol. 2022;35: 1449–1457.

15. Egevad L, Micoli C, Delahunt B, Samaratunga H, Orrason AW, Garmo H, et al. Prognosis of Gleason score 8 prostatic adenocarcinoma in needle biopsies: a nationwide population-based study. Virchows Arch. 2024. doi:10.1007/s00428-024-03810-y

16. Egevad L, Micoli C, Samaratunga H, Delahunt B, Garmo H, Stattin P, et al. Prognosis of Gleason Score 9–10 Prostatic Adenocarcinoma in Needle Biopsies: A Nationwide Population-based Study. European Urology Oncology. 2024;7: 213–221.

17. Smith R. An Overview of the Tesseract OCR Engine. Ninth International Conference on Document Analysis and Recognition (ICDAR 2007). IEEE; 2007. pp. 629–633.

18. The pandas development team. pandas-dev/pandas: Pandas. Zenodo; 2024. doi:10.5281/ZENODO.3509134
