# Supplementary material for: Development and retrospective validation of an artificial intelligence system for diagnostic assessment of prostate biopsies: study protocol
Source: BMJ Open. 2025 Jul 7;15(7):e097591. doi: 10.1136/bmjopen-2024-097591 (PMC12258300; doi:10.1136/bmjopen-2024-097591)
Supplement: online supplemental file 2 [file bmjopen-15-7-s002.pdf]

Study Protocol: Development and Retrospective Validation of an Artificial Intelligence System for Diagnostic Assessment of Prostate Biopsies

Supplementary Appendix 2

Nita Mulliqi[1], Anders Blilie[2,3], Xiaoyi Ji[1], Kelvin Szolnoky[1], Henrik Olsson[1], Matteo Titus[1], Geraldine Martinez Gonzalez[1], Sol Erika Boman[1,4], Masi Valkonen[5], Einar Gudlaugsson[2], Svein R. Kjosavik[3,6], José Asenjo[7], Marcello Gambacorta[8], Paolo Libretti[8], Marcin Braun[9], Radzislaw Kordek[9], Roman Łowicki[10], Kristina Hotakainen[11,12], Päivi Väre[13], Bodil Ginnerup Pedersen[14,15], Karina Dalsgaard Sørensen[15,16], Benedicte Parm Ulhøi[17], Mattias Rantalainen[1], Pekka Ruusuvuori[4,18], Brett Delahunt[19], Hemamali Samaratunga[20], Toyonori Tsuzuki[21], Emilius A.M. Janssen[2,22], Lars Egevad[23], Kimmo Kartasalo[24], Martin Eklund[1]

1. Department of Medical Epidemiology and Biostatistics, Karolinska Institutet, Stockholm, Sweden
2. Department of Pathology, Stavanger University Hospital, Stavanger, Norway
3. Faculty of Health Sciences, University of Stavanger, Stavanger, Norway
4. Department of Molecular Medicine and Surgery, Karolinska Institutet, Stockholm, Sweden
5. Institute of Biomedicine, University of Turku, Turku, Finland
6. The General Practice and Care Coordination Research Group, Stavanger University Hospital, Norway
7. Department of Pathology, Synlab, Madrid, Spain
8. Department of Pathology, Synlab, Brescia, Italy
9. Department of Pathology, Chair of Oncology, Medical University of Lodz, Lodz, Poland
10. 1st Department of Urology, Medical University of Lodz, Lodz, Poland
11. Department of Clinical Chemistry, University of Helsinki, Helsinki, Finland
12. Laboratory Services, Mehiläinen Oy, Helsinki, Finland
13. Mehiläinen Länsi-Pohja Hospital, Kemi, Finland
14. Department of Radiology, Aarhus University Hospital, Aarhus, Denmark
15. Department of Clinical Medicine, Aarhus University, Aarhus, Denmark
16. Department of Molecular Medicine, Aarhus University Hospital, Aarhus, Denmark
17. Department of Pathology, Aarhus University Hospital, Aarhus, Denmark
18. Faculty of Medicine and Health Technology, Tampere University, Tampere, Finland
19. Department of Pathology and Molecular Medicine, Wellington School of Medicine and Health Sciences, University of Otago, Wellington, New Zealand
20. Aquesta Uropathology and University of Queensland, QLD, Brisbane, Australia
21. Department of Surgical Pathology, School of Medicine, Aichi Medical University, Nagoya, Japan
22. Faculty of Science and Technology, University of Stavanger, Stavanger, Norway
23. Department of Oncology and Pathology, Karolinska Institutet, Stockholm, Sweden
24. Department of Medical Epidemiology and Biostatistics, SciLifeLab, Karolinska Institutet, Stockholm, Sweden

1. DATA COHORTS **2**
Development, tuning and internal validation data cohorts 2
Karolinska University Hospital (KUH-1) 2
Radboud University Medical Center (RUMC) 2
Capio S:t Göran Hospital (STG) 3
Stockholm3 (STHLM3) 4
Stavanger University Hospital (SUH) 6
External validation cohorts 7
Aichi Medical University (AMU) 7
Aquesta Uropathology morphological subtypes (AQ) 7
Aarhus University Hospital (AUH) 8
Karolinska University Hospital morphological subtypes (KUH-2) 8
Mehiläinen Länsi-Pohja (MLP) 8
Medical University of Lodz (MUL) 9
Synlab Switzerland (SCH) 10
Synlab Finland (SFI) 10
Synlab France (SFR) 11
Spear Prostate Biopsy 2020 (SPROB20) 11
University Hospital Cologne (UKK) 12
Hospital Wiener Neustadt (WNS) 12
2. TABLES **14**
3. CONSORT DIAGRAMS **22**
4. REFERENCES **40**

1. DATA COHORTS

Here, we present data cohorts used for developing and internally and externally validating the AI system. We provide details on patient selection, biopsy acquisition, histopathological sample preparation, and slide digitisation for each cohort. Additionally, we outline the reference standard protocols, specifying the variables assessed by pathologists (e.g., Gleason score (GS), International Society of Urological Pathology (ISUP) grade, perineural invasion (PNI), cribriform cancer morphology, immunohistochemistry (IHC) staining, etc.), the level of assessment (pixels, slides, anatomical locations, or patients), and any subsequent re-assessments.

Development, tuning and internal validation data cohorts

Karolinska University Hospital (KUH-1)

The KUH-1 samples were collected at the Department of Pathology, Karolinska University Hospital in Solna, Sweden in 2018. Among the cases assessed by L.E. during 2018, we included all positive slides of all patients diagnosed with ISUP grade 2-5 cancer, all positive slides from a random selection of patients diagnosed with ISUP grade 1 cancer, and all slides from a random selection of patients with a negative diagnosis. Patients underwent systematic transrectal biopsies in approximately 1/3 of the cases, and magnetic resonance imaging (MRI) targeted or combined biopsies in approximately 2/3 of cases. Slides typically contain one core, sectioned at two levels. This cohort has been used as an external validation set in previous studies [1,2].

Reference standard protocol

All cases were assessed by the lead pathologist (L.E.) using a microscope to determine the GS and cancer extent per slide, as well as the ISUP grade per slide and per patient. The linear cancer extent was generally measured from end to end in cases with discontinuous cancer and it was reported on a per-cut level.

Radboud University Medical Center (RUMC)

The RUMC samples were collected at the Radboud University Medical Center in Nijmegen, the Netherlands from January 2012 to December 2017 [3]. Patients were sampled randomly, stratified by the highest reported GS in the pathology reports, and the slide with the most aggressive part of the tumour was included for each patient. Additionally, a group of patients with only benign biopsies were randomly sampled. Patients generally underwent MRI-targeted transrectal biopsy. The data underwent additional refinement in preparation for the PANDA Kaggle challenge [2]: only one core, sectioned at one level was retained per WSI, the background was masked to hide most of the markings made on the glass, and the images were converted into .tiff format (JPEG compression, quality 70). For the purposes of PANDA, the

cohort was partitioned into three sets—development, tuning, and internal validation, stratified by patient and the highest Gleason pattern in the biopsy.

Reference standard protocol

The reference standard for all cases on the RUMC development set was determined based on the original pathology reports. Due to each slide containing multiple biopsy cores, trained non-experts digitally outlined the individual cores, allowing them to be partitioned into separate WSIs, and assigned core-level GS based on the pathology reports. Inconclusive pathology reports were assigned for a second review, and if no match could be made these cases were discarded [3].

Subsets of the cohort underwent additional re-assessments as follows:

- The PANDA RUMC tuning set (n=195, corresponds to our RUMC tuning set) and the PANDA RUMC internal validation set (n=333, part of our RUMC internal validation set) were assessed in three rounds. In the first round, three uropathologists individually graded the cases digitally, providing a GS per slide. A majority vote was taken for cases where an agreement was reached on the ISUP grade but there was a discrepancy in the Gleason patterns, and cases where two uropathologists agreed and the third one had a maximum deviation of one ISUP grade. In the second round, all the cases that did not achieve consensus were re-graded by the uropathologist whose grade differed from the others, followed by pooling of all the assessments and discussion in a consensus meeting in the third round. The GS was reported per slide.
- A subset of slides (n=66) from the RUMC internal validation cohort was randomly selected, stratified by the ISUP grade, for re-assessment by the lead pathologist (L.E.). This re-assessment was conducted digitally on Cytomine [4] using 3DHISTECH WSIs (.mrxs converted to .tiff) to report the GS per slide.

Capio S:t Göran Hospital (STG)

The STG samples were collected at Capio S:t Göran Hospital in Stockholm, Sweden from 2016 to 2017. We included a random selection of slides with an enrichment for high-grade cancer. Patients underwent transrectal biopsy, and slides typically contain one core, sectioned at two levels. This cohort was also part of the development set in a previous study [1].

Reference standard protocol

All cases were assessed by the lead pathologist (L.E.) using a microscope to provide GS, ISUP grade, and cancer extent on a per-slide level. The linear cancer extent was generally measured from end to end in cases with discontinuous cancer and it was reported on a per-cut level.

Stockholm3 (STHLM3)

The STHLM3 samples were collected in a population-based clinical trial (ISRCTN84445406) [5] from 2012 to 2015 in Stockholm, Sweden. Histological sample preparation was performed at Histocenter, Gothenburg, Sweden, and the samples were assessed at the Department of Pathology, Karolinska University Hospital in Stockholm. Patients underwent 10-12 core systematic transrectal biopsies and slides usually contain one core, sectioned at two levels. Subsets of the digitised samples have been used as development and internal validation sets in previous studies [1,2,6–8]. Patient and slide selection, retrieval and digitisation took place on five occasions between 2014 and 2023 (see Table B), as below:

- 2014: All cores from the first 500 patients diagnosed with prostate cancer in the STHLM3 trial were scanned on a Hamamatsu NanoZoomer 2.0-HT.
- 2017-2019: All patients with at least one core graded as GS 4 + 4 or 5 + 5 and 497 randomly selected patients with at least one core graded as 3 + 3 were considered. From each of these patients, we included all positive cores and a randomly selected negative core. Finally, we randomly selected 139 cancer-free patients from whom we included one randomly selected core. Additionally, we added all cores which were indicated to have PNI and had not been scanned earlier. The cores were scanned on an Aperio AT2.
- 2018-2019: The cores of a random selection of patients were scanned on a Hamamatsu NanoZoomer XR.
- 2019-2020: The cores of a random selection of patients were scanned on the Philips IntelliSite Ultra Fast Scanner (UFS).
- 2023: Patients belonging to the PANDA challenge Swedish public and private validation sets were scanned on the Grundium Ocus40.
- 2023: Initially, cores with < 4 millimetres of cancer were excluded to have sufficient cancer tissue for future molecular profiling of the samples. Among the remaining patients, 50% of those with ISUP 1 or ISUP 2 (patient level ISUP) were randomly selected for inclusion, while all patients with ISUP 3-5 were included for scanning on the Grundium Ocus40.

Reference standard protocol

All cases were assessed by the lead pathologist (L.E.) using a microscope to obtain the GS, the ISUP grade, cancer extent and PNI on a per-slide level. The linear cancer extent was generally measured from end to end in cases with discontinuous cancer and reported on a per-cut level. However, in cases with 1 or 2 cores infiltrated by low-grade discontinuous cancer with a benign gap exceeding 3 millimetres, the benign tissue was subtracted in the reporting of total cancer extent.

Subsets of the cohort underwent additional re-assessments as follows:

- A subset of slides (n=212) from the STHLM3 internal validation cohort underwent a second review to construct a reference standard for the PANDA Swedish internal

validation set. Slides initially indicated as benign according to the original reference standard were not re-reviewed, while cases indicated as malignant were divided between two uropathologists (B.D. and H.S.), each reviewing 100 slides blinded to the original review. In the case of agreement between the initial and the second review, the consensus ISUP grade was assigned to the case. In case of disagreement, a third uropathologist (T.T.) reviewed the case. For cases that were indicated as malignant by all pathologists, the final ISUP grade was assigned according to 2/3 consensus. If all three reviews were in disagreement, the case was excluded from the internal validation set. Any cases indicated as benign in the second or third review were excluded from the PANDA Swedish internal validation set. The re-assessment was conducted digitally on Cytomine using Hamamatsu and Aperio WSIs (.ndpi and .svs converted to .tiff) as described in an earlier study [2].

- A subset of slides (n=24) from the STHLM3 internal validation cohort was additionally assessed by the lead pathologist (L.E.) for specific rare morphologies (see Table E) using a microscope. This set has been used as validation data in a previous study [7].
- A subset of slides (n=87) from the STHLM3 internal validation cohort, representing the ImageBase set [9] was additionally assessed by an expert panel of uropathologists (n=23). The assessment was conducted using digital micrographs. This set has been previously used as an internal validation set in a previous study [1].
- A subset of slides (n=702) from the STHLM3 development and internal validation cohorts was digitally assessed for cribriform cancer as described in an earlier study [10]. To arrive at this selection, we first enriched Gleason pattern 4 tissue by randomly selecting one core per combination of patient and ISUP grade among all cores with ISUP grades 3-5. To maintain some representation of GS 3+4 biopsies, we randomly selected 86 additional cores with one core per patient from the set of all cores with ISUP grade 2. The slides were assessed by the lead pathologist (L.E.) on Cytomine using Hamamatsu (.ndpi) and Aperio (.svs) WSIs to create pixel-wise annotations of areas with cribriform cancer. The pathologist could also indicate uncertain cases with a borderline category.
- A subset of slides positive for cribriform cancer (n=152) and a random selection of slides negative for cribriform cancer (n=152) according to the assessment by L.E. were additionally assessed by an expert panel of uropathologists (n=9) as described in a previous study [10]. The pathologists assessed the presence of cribriform cancer on slide level on Cytomine using Hamamatsu (.ndpi) and Aperio (.svs) WSIs. The pathologists were blinded to the distribution of positive or negative slides and each other's assessments.
- All slides positive for PNI (n=485) in the STHLM3 development and internal validation cohorts were digitally re-assessed as described in [6]. The slides were assessed by the lead pathologist (L.E.) in QuPath [11] using Hamamatsu (.ndpi) and Aperio (.svs) WSIs to create pixel-wise annotations of areas of PNI.
- A subset of slides positive for PNI (n=106) and a random selection of slides negative for PNI (n=106) according to the assessment by L.E. was additionally assessed by an expert

panel of uropathologists (n=4) as described in a previous study [12]. The pathologists assessed the presence of PNI on slide level on Cytomine using Hamamatsu (.ndpi) and Aperio (.svs) WSIs. The pathologists were blinded to the distribution of positive or negative slides and to each other's assessments. The pathologists could also indicate uncertain cases with borderline categories.

Stavanger University Hospital (SUH)

The SUH samples represent consecutive cases collected from routine diagnostics at the Department of Pathology, Stavanger University Hospital in Stavanger, Norway from December 2016 to March 2018. Biopsies were taken at the Department of Urology in Stavanger University Hospital and other private urological clinics at the Stavanger Urological Center. Patients primarily underwent systematic transrectal biopsies, although some received MRI-targeted biopsies, either alone or combined with systematic biopsy. Slides typically contain two cores from the same anatomical location, sectioned at two levels. A subset of the SUH cohort has been used as an external validation set in previous studies [7,8].

Reference standard protocol

The reference standard was obtained from the original pathology reports from the clinical routine. Seven uropathologists and seven general pathologists assessed the slides microscopically reporting the GS, ISUP grade, Gleason pattern 4 percentage, cancer extent, biopsy length, PNI, fatty tissue infiltration (FTI), and additional stainings (e.g. IHC) on the slide level. The linear cancer extent was generally measured from end to end in cases with discontinuous cancer and it was reported on a per-cut level.

Subsets of the SUH cohort underwent additional re-assessments as follows:

- A subset of slides (n=66) from the SUH internal validation cohort was randomly selected and stratified by ISUP grade for re-assessment by the lead pathologist (L.E.). This re-assessment was conducted digitally on Cytomine using Hamamatsu WSIs (.ndpi) to report the GS per slide.
- A subset of slides (n=332) with Gleason pattern 4 tissue from the SUH development and internal validation cohorts was initially assessed by a uropathologist (A.B.) for potential cribriform cancer using QuPath. We then randomly selected at most 90 positive, 30 borderline and 30 negative slides from the development cohort and at most 30 positive, 10 borderline and 10 negative slides from the internal validation cohort to be re-assessed by the lead pathologist (L.E.), resulting in 200 slides. This re-assessment was conducted digitally on Cytomine using Hamamatsu (.ndpi) WSIs to report cribriform cancer per slide. The pathologist could also indicate uncertain cases with a borderline category.
- All slides from cases reported as positive for PNI in the SUH development and internal validation cohorts were initially assessed by a uropathologist (A.B.) for potential PNI using a microscope. We then randomly selected at most 25 positive and 5 negative slides

per ISUP grade from the development cohort, and at most 8 positive and 2 negative slides per ISUP grade from the internal validation cohort to be re-assessed by the lead pathologist (L.E.), resulting in 185 slides. This re-assessment was conducted digitally on Cytomine using Hamamatsu (.ndpi) WSIs to report PNI per slide. The pathologist could also indicate uncertain cases with a borderline category.

External validation cohorts

Aichi Medical University (AMU)

The AMU samples were collected at the Aichi Medical University in Nagakute, Japan from 2020 to 2023. Samples were selected to include cribriform prostate cancer cases and non-cribriform cases. Cribriform cases were chosen sequentially, while non-cribriform cases were selected among cases containing Gleason pattern 4 and age-adjusted to match the cribriform cases. Patients generally underwent systematic transrectal biopsy, with only a few undergoing MRI-targeted biopsy. Slides typically contain several cores, sectioned at several levels.

Reference standard protocol

All cases were assessed by a uropathologist (T.T.) initially using a microscope and then confirmed digitally with the NDP.View software using Hamamatsu WSIs (.ndpi). The presence or absence of cribriform prostate cancer was reported on slide level and GS was reported on patient level.

Aquesta Uropathology morphological subtypes (AQ)

The AQ cases were collected at the Aquesta Specialised Uropathology laboratory in Toowong, Australia from 2009 to 2023. The biopsies were performed in private hospitals and urology clinics in Queensland state, Australia. Slides were specifically selected to represent rare morphologies such as benign mimickers of prostate cancer which are typically hard to diagnose in routine pathology. Patients generally underwent MRI-targeted transrectal biopsies, and each slide has two cores, sectioned at two levels.

Reference standard protocol

A uropathologist (H.S.) assessed the slides microscopically and reported the GS, ISUP grade, additional stainings (e.g. IHC), and the presence or absence of specific morphological subtype categories on slide level (see Table E). Slides representing benign mimickers were microscopically re-assessed by the lead pathologist (L.E.).

Aarhus University Hospital (AUH)

The AUH samples were part of the PRIMA clinical trial conducted at the Aarhus University Hospital in Aarhus, Denmark from January 2018 to December 2021 [13]. Histopathology assessment was conducted at the Department of Pathology, Aarhus University Hospital, Aarhus, Denmark. In this trial, men aged 50-59 years with elevated prostate-specific antigen (PSA) (3-10 ng/ml) and/or positive STHLM3 test (defined as STHLM3 score equal to or above 11%) and MRI of PIRADS 3-5 were referred to MRI-targeted transrectal biopsy. Out of 117 patients who underwent the biopsy procedure, the pathologist selected slides based on histopathological features with the aim of a uniform distribution of ISUP grades. Slides typically contain two cores, sectioned at three levels. This cohort was used as an external validation set in a previous study [8].

Reference standard protocol

All cases were assessed by a uropathologist (B.P.U.) microscopically and the GS, the ISUP grade, cancer extent and biopsy length were reported on the slide level.

Subsets of the AUH cohort underwent additional re-assessments as follows:

- A subset of slides (n=41) was randomly selected, stratified by the ISUP grade, for re-assessment by the lead pathologist (L.E.). This re-assessment was conducted digitally on Cytomine using Hamamatsu WSIs (.ndpi) to report the GS per slide.

Karolinska University Hospital morphological subtypes (KUH-2)

The KUH-2 samples were collected at the Department of Pathology, Karolinska University Hospital in Solna, Sweden in 2022. The biopsy procedure and number of tissue sections per slide adhere to the KUH-1 cohort. Similarly to the AQ cohort, these samples were specifically selected to represent cases that are typically challenging to diagnose in clinical practice, such as rare disease morphologies and benign mimickers. This cohort was used as an external validation set in a previous study [7].

Reference standard protocol

The reference standard protocol for the KUH-2 cohort adheres to KUH-1, except for additional reporting of the presence or absence of specific morphological subtype categories, assessed by the lead pathologist (L.E.) on slide level (see Table E).

Mehiläinen Länsi-Pohja (MLP)

The MLP samples represent consecutive cases from routine pathology at the Mehiläinen Länsi-Pohja Hospital in Kemi, Finland from 2016 to 2019. Patients underwent systematic transrectal biopsies, and biopsies were sampled based on anatomical location: left and right

typically consisting of six cores per location. Slides typically contain one core, sectioned at two to three levels.

Reference standard protocol

The reference standard was obtained from routine assessments done by several pathologists using a microscope to determine the GS, the ISUP grade, cancer extent and biopsy length per patient or per anatomical location (i.e. a set of biopsy cores assessed together).

Subsets of the MLP cohort underwent additional re-assessments as follows:

- A subset of slides (n=66) was randomly selected, stratified by the ISUP grade, for re-assessment by the lead pathologist (L.E.). The patient level ISUP grade was used for stratification, due to missing slide level grading. This re-assessment was conducted digitally on Cytomine using 3DHISTECH WSIs (.mrxs) to report the GS per slide.

Medical University of Lodz (MUL)

The MUL samples represent consecutive cases from routine pathology at the 1st Department of Urology, University Clinical Hospital of the Military Academy of Medicine - Central Veterans Hospital, Medical University of Lodz, Lodz, Poland from January 2018 to March 2019. Histopathological assessment was conducted at the Department of Pathology, Department of Oncology, Medical University of Lodz, Lodz, Poland. Patients underwent systematic transrectal biopsy and slides typically contain one core, sectioned at four to seven levels.

Reference standard protocol

The reference standard was determined based on an initial assessment by a single pathologist (M.B.) and a second review by a more experienced pathologist (R.K.). Both pathologists have a specialisation in surgical pathology and are currently specialising in uropathology. The pathologists assessed the cases using a microscope and reported the GS, the ISUP grade, total cancer percentage and Gleason pattern 4 and 5 percentages on the slide level.

Subsets of the MUL cohort underwent additional re-assessments as follows:

- A subset of slides (n=66) was randomly selected, stratified by ISUP grade, for re-assessment by the lead pathologist (L.E.). This re-assessment was conducted digitally on Cytomine using Grundium WSIs (.svs) to report the GS per slide.
- All slides containing Gleason pattern 4 (n=276) were initially assessed for potential cribriform cancer by a uropathologist (A.B.). The assessment was conducted digitally on Cytomine using Grundium WSIs (.svs) to report cribriform cancer per slide and mark the positive and borderline foci. All foci were then re-assessed on Cytomine by the lead pathologist (L.E.).

- The slides (n=276) assessed for cribriform cancer were also initially assessed for potential PNI by a uropathologist (A.B.). The assessment was conducted on Cytomine using Grundium WSIs (.svs) to report PNI per slide and mark the positive and borderline foci. All foci were then re-assessed on Cytomine by the lead pathologist (L.E.).

Synlab Switzerland (SCH)

The SCH samples represent consecutive cases from routine diagnoses at the Argot Laboratory in Lausanne, Switzerland from January 2020 to December 2020. Patients underwent systematic, MRI-targeted or combined transrectal biopsies. Slides typically contain one core, sectioned at two levels. A varying number of cores were typically obtained from a varying number of anatomical locations.

Reference standard protocol

The reference standard was determined based on the pathology reports from routine diagnostics. Using the microscope the pathologists reported the GS, the ISUP grade, cancer extent, biopsy length, Gleason pattern 4 percentage, cribriform cancer, PNI, high-grade prostatic intraepithelial neoplasia (HGPIN) and possible IHC staining per anatomical location (i.e. a set of biopsy cores assessed together) and per patient.

Subsets of the SCH cohort underwent additional re-assessments as follows:

- A subset of slides (n=72) was randomly selected, stratified by the ISUP grade and anatomical location for re-assessment by the lead pathologist (L.E.). This re-assessment was conducted digitally on Cytomine using Philips WSIs (.isyntax converted to .tiff) to report the GS per slide.
- A subset of slides (n=56) were digitally re-assessed for cribriform cancer by a uropathologist (H.S.). We selected all positive anatomical locations and a random selection of 6 negative anatomical locations with Gleason pattern 4 tissue and included all slides from these locations. This re-assessment was conducted digitally on Cytomine using Philips WSIs (.isyntax converted to .tiff) to report cribriform cancer per slide. The pathologist could also indicate uncertain cases with a borderline category.
- A subset of slides (n=94) were digitally re-assessed for PNI by a uropathologist (B.D.). We randomly selected 12 positive and 5 negative anatomical locations per ISUP grade and included all slides from these locations. This re-assessment was conducted digitally on Cytomine using Philips WSIs (.isyntax converted to .tiff) to report PNI per slide. The pathologist could also indicate uncertain cases with a borderline category.

Synlab Finland (SFI)

The SFI samples represent consecutive cases from routine diagnostics at the Synlab Laboratory in Helsinki, Finland from January 2020 to February 2021. Patients underwent systematic,

MRI-targeted or combined transrectal biopsies. Slides typically contain two cores, sectioned at five to six levels. A varying number of cores were typically obtained from a varying number of anatomical locations.

Reference standard protocol

The reference standard was determined based on the pathology reports from routine diagnostics. Using the microscope the pathologists reported the GS, the ISUP grade, cancer extent, biopsy length, Gleason pattern 4 percentage, cribriform cancer, PNI, HGPIN and possible IHC staining per anatomical location (i.e. a set of biopsy cores assessed together) and in some cases per patient.

Subsets of the SFI cohort underwent additional re-assessments as follows:

- A subset of slides (n=67) was randomly selected, stratified by the ISUP grade and anatomical location for re-assessment by the lead pathologist (L.E.). This re-assessment was conducted digitally on Cytomine using Philips WSIs (.isyntax converted to .tiff) to report the GS per slide.

Synlab France (SFR)

The SFR samples represent consecutive cases from routine diagnostics at the Technipath-Synlab Medical Laboratory in Dommartin, Rhône, France from September 2020 to December 2020. Patients underwent systematic, MRI-targeted or combined transrectal biopsies. Slides usually contain two to three cores from the same anatomical location, sectioned at two levels.

Reference standard protocol

The reference standard was determined based on the pathology reports from routine diagnostics. Pathologists using a microscope reported the GS, the ISUP grade, cancer extent, biopsy length, Gleason pattern 4 percentage, cribriform cancer, PNI, HGPIN and possible IHC staining per anatomical location (i.e. slide) and in some cases per patient.

Subsets of the SFR cohort underwent additional re-assessments as follows:

- A subset of slides (n=49) was randomly selected, stratified by the ISUP grade and anatomical location for re-assessment by the lead pathologist (L.E.). This re-assessment was conducted digitally on Cytomine using Philips WSIs (.isyntax converted to .tiff) to report the GS per slide.

Spear Prostate Biopsy 2020 (SPROB20)

The SPROB20 samples were collected at Uppsala University Hospital, Uppsala, Sweden from 2015 to 2018. Patients underwent targeted transrectal biopsies. Slides typically contain one core, sectioned at one level. This cohort is publicly available at the AIDA Data Hub [14].

Reference standard protocol

The reference standard was obtained from the clinical routine. The pathologists assessed the slides microscopically and reported the ISUP grade at the patient level in two ways: as the maximum and as the average of the slide level ISUP grades. The underlying slide-level ISUP grades were not provided on the AIDA Data Hub.

Subsets of the SPROB20 cohort underwent additional re-assessments as follows:

- A subset of slides (n=50) was randomly selected, stratified by ISUP grade and patient, for re-assessment by the lead pathologist (L.E.). This re-assessment was conducted digitally on Cytomine using Hamamatsu WSIs (.ndpi converted to .tiff) to report the GS per slide.

University Hospital Cologne (UKK)

The UKK samples represent consecutive cases from the Institute of Pathology at the University Hospital Cologne in Cologne, Germany. Patients underwent combined systematic and MRI-targeted transrectal biopsies. Slides typically contain one core, sectioned at three levels. The publicly available subset of samples was randomly selected and stratified by the ISUP grade, including ten samples per ISUP grade. This cohort was obtained from a publicly available dataset which was part of the development and validation sets in an earlier study [15]. The WSIs were converted from JPEG2000 compressed OME-TIFF format via an intermediate raw Zarr format to JPEG compressed (quality 80) generic pyramidal TIFF format for OpenSlide compatibility using the *bioformats2raw* (v. 0.9.3), *raw2ometiff* (v. 0.7.1) and *libvips* (v. 8.9.1) converters.

Reference standard protocol

The reference standard was determined digitally by a panel of 10 different pathologists from Austria, Germany, Israel, Japan, the Netherlands, Russia and the United States. All pathologists reported the ISUP grade per slide and the final grade was obtained as the majority vote. A consensus was considered reached in cases where the majority ISUP grade had at least six votes.

Hospital Wiener Neustadt (WNS)

The WNS samples represent consecutive cases from the Hospital Wiener Neustadt in Wiener Neustadt, Austria. Patients underwent combined systematic and MRI-targeted transrectal biopsies. Slides typically contain one core, sectioned at one level. The publicly available subset of samples was randomly selected and stratified by the ISUP grade, including ten samples per ISUP grade. This cohort was obtained from a publicly available dataset which was part of the development and validation sets in an earlier study [15]. The WSIs were converted from JPEG2000 compressed OME-TIFF format via an intermediate raw Zarr format to JPEG compressed (quality 80) generic pyramidal TIFF format for OpenSlide compatibility using the *bioformats2raw* (v. 0.9.3), *raw2ometiff* (v. 0.7.1) and *libvips* (v. 8.9.1) converters.

Reference standard protocol

The reference standard was determined digitally by a panel of 11 different pathologists from Austria, Germany, Israel, Japan, the Netherlands, Russia and the United States. All pathologists reported the ISUP grade per slide and the final grade was obtained as the majority vote. A consensus was considered reached in cases where the majority ISUP grade had at least six votes.

2. TABLES

Table 1. Patient clinical and pathological characteristics. Patient and slide level information for the development, tuning, internal, and external validation cohorts including age, PSA, ISUP grade and cancer length distributions. Averaged age and PSA are shown for patients who underwent multiple biopsies. The ISUP distributions are based on the initial, original grading excluding any re-assessments. For the AMU, MLP, SCH, SFI, SFR and SPROB20 cohorts, where pathology reporting was performed on anatomical location or patient level, the total summed numbers of slides associated with a given ISUP grade or cancer length are shown. The AUH cohort has an age range of 50.4 to 69.9 yrs (mean 63.2 yrs, median 64.0 yrs) and a PSA range of 1.5 ng/mL to 9.8 ng/mL (mean 4.6 ng/mL, median 4.2 ng/mL). The SPROB20 cohort has an age range of 39 to 79 yrs (median 67 yrs). Slides in the AQ, KUH-2 and SUH cohorts missing ISUP grade information represent non-gradable morphological variants. PSA=prostate-specific antigen, ISUP=International Society of Urological Pathology, STHLM3=Stockholm3, SUH=Stavanger University Hospital, RUMC=Radboud University Medical Center, STG=Capio S:t Göran Hospital, KUH-1=Karolinska University Hospital, AMU=Aichi Medical University, AQ=Aquesta Uropathology, AUH=Aarhus University Hospital, KUH-2=Karolinska University Hospital morphological subtypes, MLP=Mehiläinen Länsi-Pohja, MUL=Medical University of Lodz, SCH=Synlab Switzerland, SFI=Synlab Finland, SFR=Synlab France, SPROB20=Spear Prostate Biopsy 2020, UKK=University Hospital Cologne, WNS=Hospital Wiener Neustadt, N/A=Not available.

**Development**	**STHLM3**	**SUH**	**RUMC**	**STG**
**No. participants (%)**	**n=2,711**	**n=710**	**n=976**	**n=70**
**Age, years**				
<=49 yrs	4 (0.14)	13 (1.83)		0 (0.0)
50 - 54 yrs	216 (7.96)	35 (4.92)		1 (1.42)
55 - 59 yrs	429 (15.82)	94 (13.23)		2 (2.85)
60 - 64 yrs	702 (25.89)	137 (19.29)	N/A	4 (5.71)
65 - 69 yrs	1,207 (44.52)	191 (26.90)		6 (8.57)
>= 70 yrs	153 (5.64)	240 (33.80)		37 (52.85)
Missing	0 (0.0)	0 (0.0)		20 (28.6)
**Prostate-specific antigen**				
<3 ng/mL	611 (22.53)	60 (8.45)		2 (2.85)
3 - <5 ng/mL	1,306 (48.17)	135 (19.01)		1 (1.42)
5 - <10 ng/mL	592 (21.83)	350 (49.29)	N/A	6 (8.57)
>= 10 ng/mL	202 (7.45)	163 (22.95)		38 (54.28)
Missing	0 (0.0)	2 (0.28)		23 (32.85)

No. slides (%)	n=29,536	n=4,606	n=4,564	n=247
**Cancer length**				
No cancer	23,530 (79.67)	3,435 (74.57)	N/A	1 (0.40)
>0 - 1 mm	2,021 (6.84)	238 (5.16)		7 (2.83)
>1 - 5 mm	2,577 (8.72)	405 (8.78)		42 (17.00)
>5 - 10 mm	1,054 (3.56)	226 (4.90)		86 (34.81)
>10 mm	354 (1.19)	300 (6.51)		111 (44.93)
Missing	0 (0.0)	2 (0.04)		0 (0.0)
**Cancer grade**				
Benign	23,530 (79.67)	3,435 (74.57)	912 (19.98)	1 (0.40)
ISUP 1 (3+3)	3,571 (12.09)	683 (14.82)	731 (16.01)	1 (0.40)
ISUP 2 (3+4)	1,265 (4.28)	240 (5.20)	594 (13.01)	1 (0.40)
ISUP 3 (4+3)	494 (1.67)	129 (2.79)	800 (17.52)	2 (0.80)
ISUP 4 (4+4, 3+5, 5+3)	377 (1.28)	54 (1.17)	668 (14.63)	32 (12.95)
ISUP 5 (4+5, 5+4, 5+5)	299 (1.01)	63 (1.36)	859 (18.82)	210 (85.02)
Missing	0 (0.0)	2 (0.04)	0 (0.0)	0 (0.0)

Internal validation	STHLM3	SUH	RUMC
**No. participants (%)**	**n=654**	**n=178**	**n=172**
**Age, years**			
<=49 yrs	3 (0.45)	1 (0.56)	N/A
50 - 54 yrs	58 (8.86)	6 (3.37)	
55 - 59 yrs	96 (14.67)	15 (8.42)	
60 - 64 yrs	182 (27.82)	39 (21.91)	
65 - 69 yrs	289 (44.18)	46 (25.84)	
>= 70 yrs	26 (3.97)	71 (39.88)	
Missing	0 (0.0)	0 (0.0)	
**Prostate-specific antigen**			
<3 ng/mL	123 (18.80)	12 (6.74)	N/A
3 - <5 ng/mL	321 (49.08)	23 (12.92)	
5 - <10 ng/mL	153 (23.39)	91 (51.12)	
>= 10 ng/mL	57 (8.71)	52 (29.21)	
Missing	0 (0.0)	0 (0.0)	

No. slides (%)	n=7,036	n=1,156	n=516
**Cancer length**			
No cancer	5,098 (72.45)	736 (63.70)	N/A
>0 - 1 mm	583 (8.28)	52 (4.48)	
>1 - 5 mm	767 (10.90)	109 (9.48)	
>5 - 10 mm	434 (6.16)	87 (7.50)	
>10 mm	154 (2.18)	172 (14.82)	
Missing	0 (0.0)	0 (0.0)	
**Cancer grade**			
Benign	5,098 (72.46)	736 (63.66)	195 (37.79)
ISUP 1 (3+3)	958 (13.62)	153 (13.23)	87 (16.86)
ISUP 2 (3+4)	380 (5.40)	76 (6.55)	45 (8.72)
ISUP 3 (4+3)	240 (3.41)	74 (6.37)	77 (14.92)
ISUP 4 (4+4, 3+5, 5+3)	203 (2.89)	53 (4.56)	54 (10.46)
ISUP 5 (4+5, 5+4, 5+5)	157 (2.23)	64 (5.51)	58 (11.24)
Missing	0 (0.0)	0 (0.0)	0 (0.0)

Tuning	STHLM3	KUH-1	RUMC
**No. participants (%)**	**n=24**	**n=73**	**n=72**
**Age, years**			
<=49 yrs	0 (0.0)	2 (2.73)	N/A
50 - 54 yrs	1 (4.16)	5 (6.84)	
55 - 59 yrs	2 (8.33)	10 (13.69)	
60 - 64 yrs	8 (33.33)	12 (16.43)	
65 - 69 yrs	13 (54.16)	15 (20.54)	
>= 70 yrs	0 (0.0)	29 (39.72)	
Missing	0 (0.0)	0 (0.0)	
**Prostate-specific antigen**			
<3 ng/mL	3 (12.50)	N/A	N/A
3 - <5 ng/mL	12 (50.00)		
5 - <10 ng/mL	5 (20.83)		
>= 10 ng/mL	4 (16.66)		
Missing	0 (0.0)		

No. slides (%)	n=276	n=330	n=195
**Cancer length**			
No cancer	192 (69.57)	108 (32.72)	N/A
>0 - 1 mm	32 (11.59)	33 (10.00)	
>1 - 5 mm	27 (9.67)	77 (23.33)	
>5 - 10 mm	16 (5.73)	75 (22.72)	
>10 mm	9 (3.22)	37 (11.21)	
Missing	0 (0.0)	0 (0.0)	
**Cancer grade**			
Benign	192 (69.57)	108 (32.72)	95 (48.72)
ISUP 1 (3+3)	28 (10.14)	65 (19.70)	24 (12.31)
ISUP 2 (3+4)	18 (6.52)	63 (19.09)	15 (7.69)
ISUP 3 (4+3)	13 (4.71)	49 (14.85)	15 (7.69)
ISUP 4 (4+4, 3+5, 5+3)	13 (4.71)	19 (5.76)	19 (9.74)
ISUP 5 (4+5, 5+4, 5+5)	12 (4.35)	26 (7.88)	27 (13.85)
Missing	0 (0.0)	0 (0.0)	0 (0.0)

External validation	AMU	AQ	AUH
**No. participants (%)**	**n=43**	**n=135**	**n=42**
**Age, years**			
<= 49 yrs	N/A	N/A	N/A
50 - 54 yrs			
55 - 59 yrs			
60 - 64 yrs			
65 - 69 yrs			
>= 70 yrs			
Missing			
**Prostate-specific antigen**			
<3 ng/mL	1 (2.32)	N/A	N/A
3 - <5 ng/mL	1 (2.32)		
5 - <10 ng/mL	11 (25.58)		
>= 10 ng/mL	30 (69.76)		
Missing	0 (0.0)		

No. slides (%)	n=73	n=136	n=102
**Cancer length**			
No cancer			43 (42.15)
>0 - 1 mm			5 (4.90)
>1 - 5 mm	N/A	N/A	18 (17.64)
>5 - 10 mm			24 (23.52)
>10 mm			12 (11.76)
Missing			0 (0.0)
**Cancer grade**			
Benign	0 (0.0)	122 (89.70)	43 (42.15)
ISUP 1 (3+3)	0 (0.0)	1 (0.73)	26 (25.49)
ISUP 2 (3+4)	0 (0.0)	1 (0.73)	25 (24.50)
ISUP 3 (4+3)	6 (8.21)	0 (0.00)	1 (0.98)
ISUP 4 (4+4, 3+5, 5+3)	22 (28.76)	0 (0.00)	7 (6.86)
ISUP 5 (4+5, 5+4, 5+5)	45 (60.27)	1 (0.73)	0 (0.0)
Missing	0 (0.0)	11 (8.08)	0 (0.0)

External validation	KUH-2	MLP	MUL
**No. participants (%)**	**n=89**	**n=199**	**n=207**
**Age, years**			
<=49 yrs			2 (0.96)
50 - 54 yrs			4 (1.93)
55 - 59 yrs			10 (4.83)
60 - 64 yrs	N/A	N/A	29 (14.00)
65 - 69 yrs			50 (24.15)
>= 70 yrs			108 (52.17)
Missing			4 (1.96)
**Prostate-specific antigen**			
<3 ng/mL		19 (9.54)	
3 - <5 ng/mL		26 (13.06)	
5 - <10 ng/mL	N/A	65 (32.66)	N/A
>= 10 ng/mL		85 (42.71)	
Missing		4 (2.03)	

No. slides (%)	n=146	n=1,964	n=1,959
**Cancer length**			
No cancer	N/A	302 (15.37)	N/A
>0 - 1 mm		24 (1.22)	
>1 - 5 mm		207 (10.53)	
>5 - 10 mm		191 (9.72)	
>10 mm		1,189 (60.53)	
Missing		54 (2.63)	
**Cancer grade**			
Benign	103 (70.54)	323 (16.44)	1,483 (75.70)
ISUP 1 (3+3)	34 (23.28)	433 (22.04)	161 (8.21)
ISUP 2 (3+4)	5 (3.42)	506 (25.76)	58 (2.96)
ISUP 3 (4+3)	0 (0.0)	216 (10.99)	74 (3.77)
ISUP 4 (4+4, 3+5, 5+3)	0 (0.0)	133 (6.77)	65 (3.31)
ISUP 5 (4+5, 5+4, 5+5)	0 (0.0)	353 (17.97)	118 (6.02)
Missing	4 (2.73)	0 (0.0)	0 (0.0)

**External validation**	**SCH**	**SFI**	**SFR**
**No. participants (%)**	**n=199**	**n=99**	**n=84**
**Age, years**			
<=49 yrs	3 (1.50)	N/A	1 (1.19)
50 - 54 yrs	3 (1.50)		5 (5.95)
55 - 59 yrs	22 (11.05)		11 (13.09)
60 - 64 yrs	27 (13.56)		11 (13.09)
65 - 69 yrs	46 (23.11)	2 (2.0)	21 (25.00)
>= 70 yrs	98 (49.24)	3 (3.03)	35 (41.66)
Missing	0 (0.0)	94 (94.97)	0 (0.0)
**Prostate-specific antigen**			
Low	0 (0.0)	2 (2.02)	0 (0.0)
Normal	0 (0.0)	2 (2.02)	0 (0.0)
Elevated	19 (9.54)	8 (8.08)	0 (0.0)
<3 ng/mL	3 (1.50)	2 (2.02)	1 (1.35)
3 - <5 ng/mL	21 (10.55)	8 (8.08)	6 (7.14)

5 - <10 ng/mL	45 (22.61)	39 (39.39)	51 (60.71)
>= 10 ng/mL	39 (19.59)	32 (32.32)	16 (19.04)
Missing	72 (36.18)	6 (6.06)	10 (11.75)
**No. slides (%)**	**n=2,434**	**n=537**	**n=515**
**Cancer length**			
No cancer	1,580 (64.91)	311 (57.91)	373 (72.42)
>0 - 1 mm	22 (0.90)	16 (2.97)	1 (0.19)
>1 - 5 mm	156 (6.39)	39 (7.26)	34 (6.60)
>5 - 10 mm	88 (3.60)	30 (5.58)	32 (6.21)
>10 mm	565 (23.27)	54 (10.05)	69 (13.39)
Missing	23 (0.94)	87 (16.42)	6 (0.97)
**Cancer grade**			
Benign	1,580 (64.91)	311 (57.91)	373 (72.42)
ISUP 1 (3+3)	325 (13.31)	61 (11.35)	87 (16.89)
ISUP 2 (3+4)	201 (8.25)	51 (9.49)	28 (5.43)
ISUP 3 (4+3)	183 (7.51)	50 (9.31)	3 (0.58)
ISUP 4 (4+4, 3+5, 5+3)	94 (3.86)	16 (2.97)	10 (1.94)
ISUP 5 (4+5, 5+4, 5+5)	47 (1.93)	30 (5.58)	6 (1.16)
Missing	4 (0.16)	18 (3.39)	8 (1.55)
**External validation**	**SPROB20**	**UKK**	**WNS**
**No. participants (%)**	**n=452**	**n=50**	**n=50**
**Age, years**			
<=49 yrs	N/A	N/A	N/A
50 - 54 yrs			
55 - 59 yrs			
60 - 64 yrs			
65 - 69 yrs			
>= 70 yrs			
Missing			
**Prostate-specific antigen**			
<3 ng/mL	13 (2.87)	N/A	N/A
3 - <5 ng/mL	14 (3.09)		

5 - <10 ng/mL	30 (6.63)		
>= 10 ng/mL	190 (42.03)		
Missing	205 (45.35)		

**No. slides (%)**	**n=2,570**	**n=50**	**n=50**
No cancer	N/A	N/A	N/A
>0 - 1 mm			
>1 - 5 mm			
>5 - 10 mm			
>10 mm			
Missing			
**Cancer grade**			
Benign	950 (36.96)	0 (0.0)	0 (0.0)
ISUP 1 (3+3)	543 (21.12)	12 (24.0)	10 (20.0)
ISUP 2 (3+4)	700 (27.23)	8 (16.0)	10 (20.0)
ISUP 3 (4+3)	186 (7.23)	12 (24.0)	12 (24.0)
ISUP 4 (4+4, 3+5, 5+3)	103 (4.00)	8 (16.0)	8 (16.0)
ISUP 5 (4+5, 5+4, 5+5)	88 (3.42)	10 (20.0)	10 (20.0)
Missing	0 (0.0)	0 (0.0)	0 (0.0)

3. CONSORT DIAGRAMS

The flow charts below represent CONSORT diagrams for all the data cohorts utilised in this study. Subsequent exclusions will be incorporated in revised versions of this protocol if any additional technical issues arise during model development and validation e.g. file corruption issues encountered during whole slide image (WSI) preprocessing or generating tiles from the WSI. CONSORT diagram figures are created with BioRender.com.

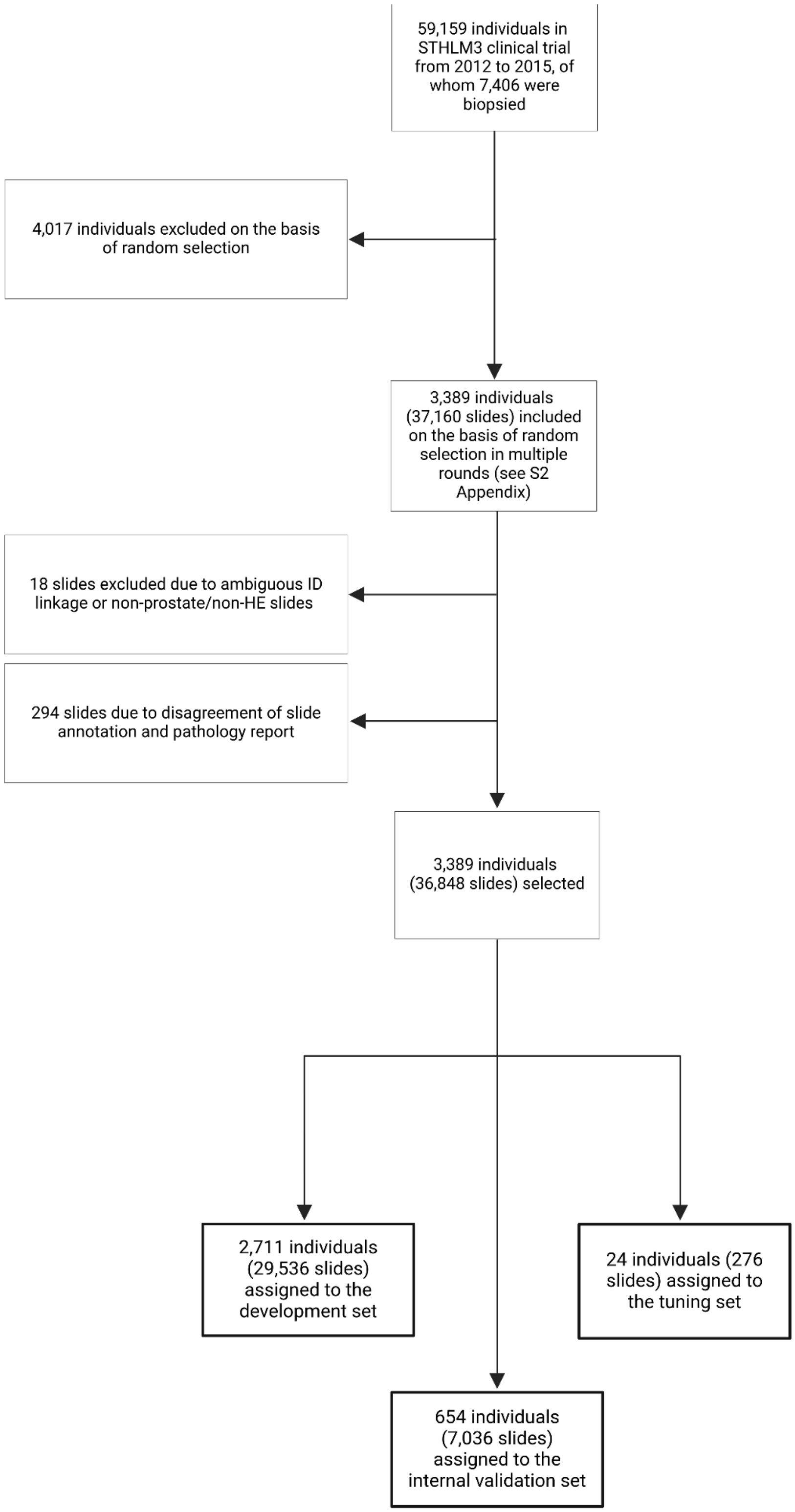

Fig S1. CONSORT diagram for the Stockholm3 (STHLM3) cohort, which is part of the development, tuning and internal validation set.

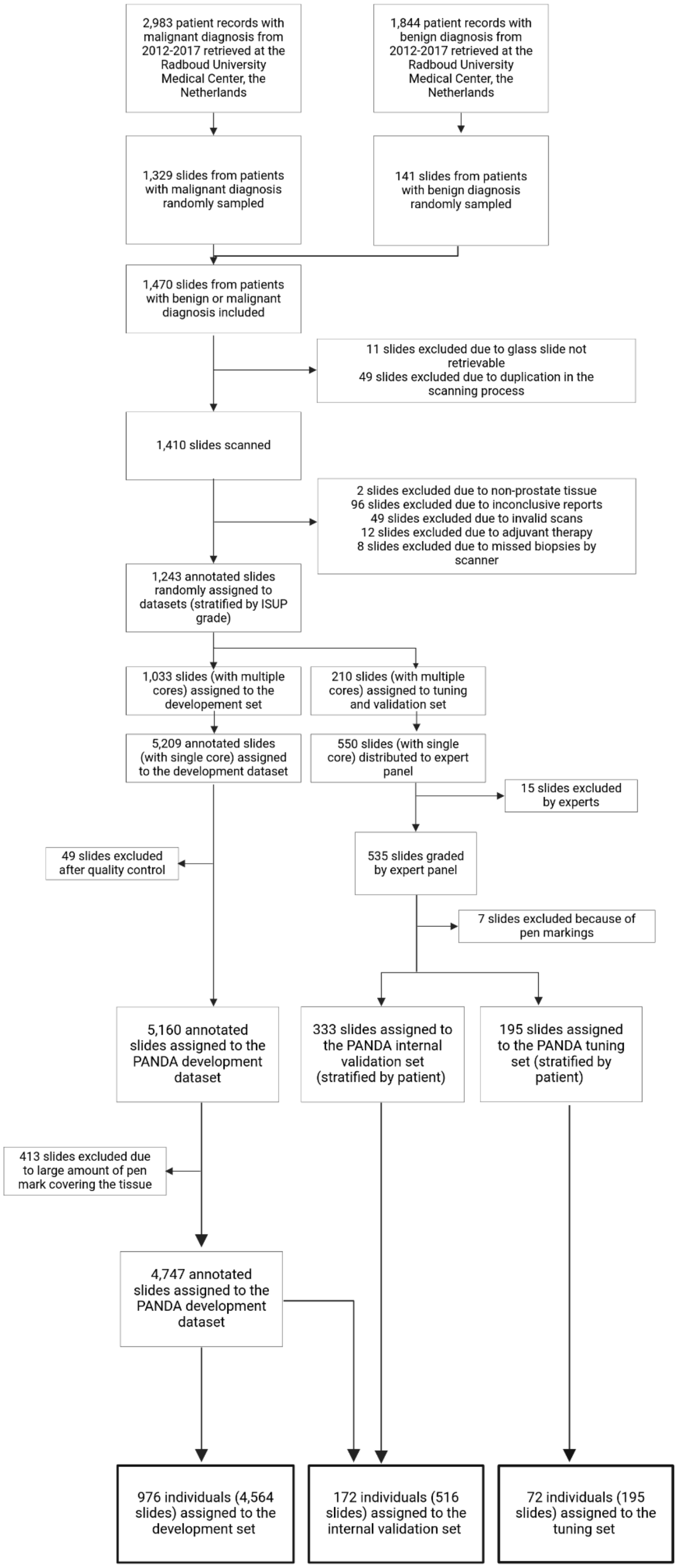

Fig S2. CONSORT diagram for the Radboud University Medical Center (RUMC) cohort, which is part of the development, tuning and internal validation set. Adapted from [2].

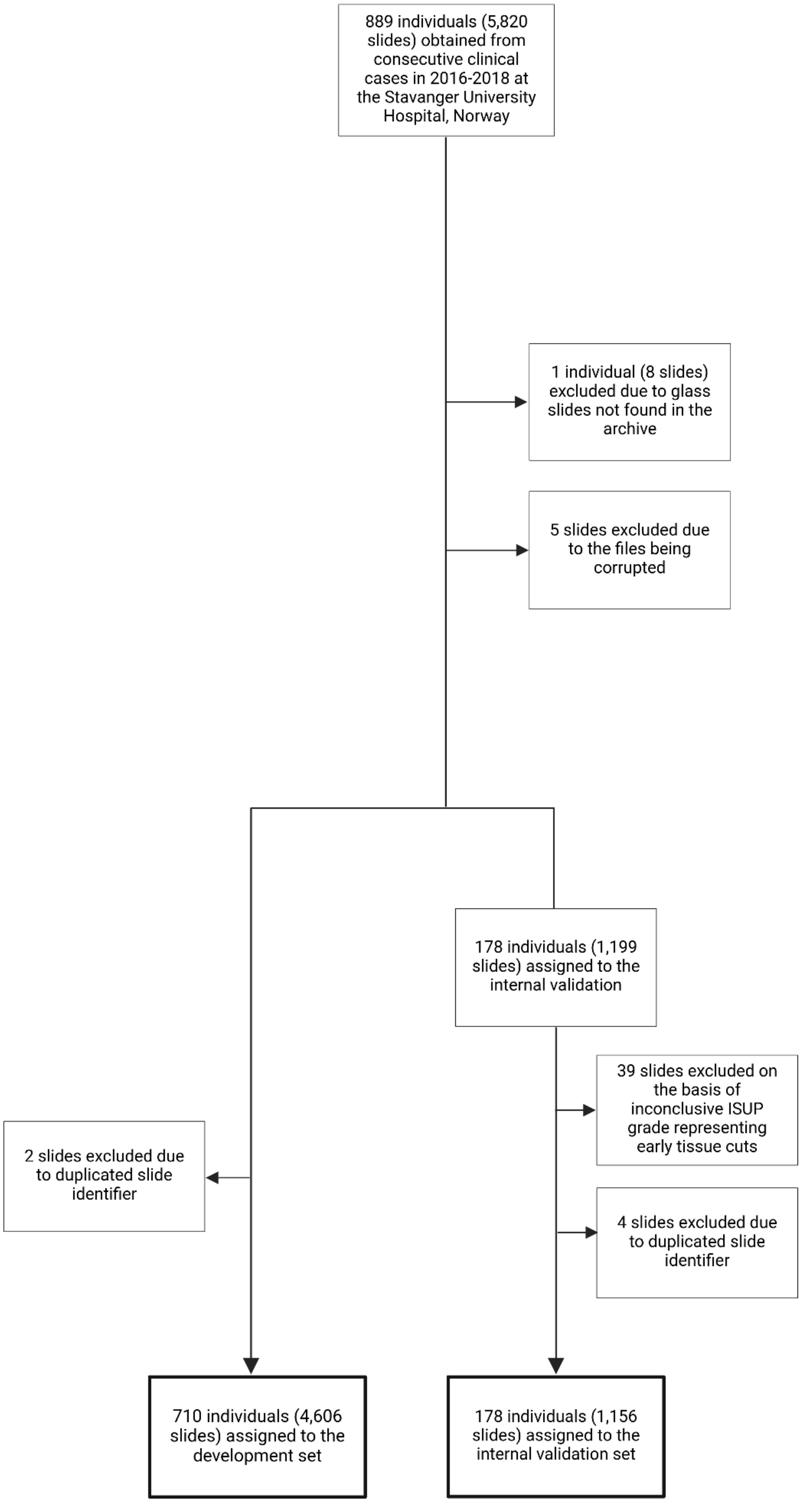

Fig S3. CONSORT diagram for the Stavanger University Hospital (SUH) cohort, which is part of the development and internal validation set.

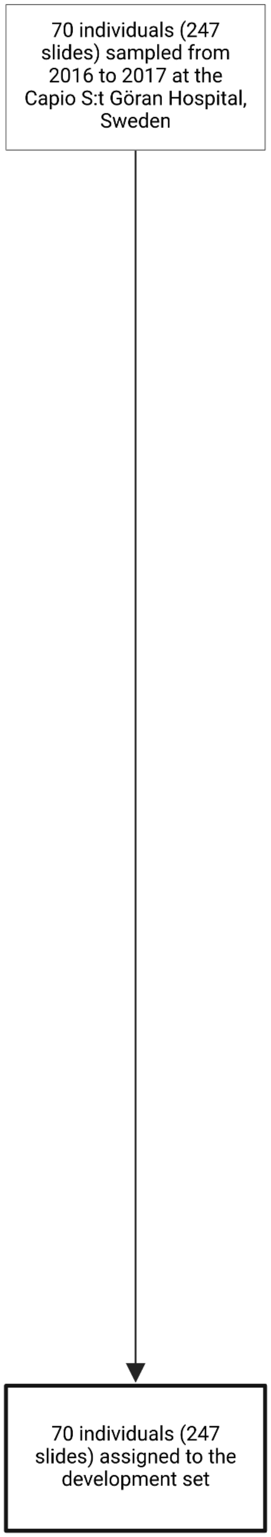

Fig S4. CONSORT diagram for the Capio S:t Göran Hospital (STG) cohort, which is part of the development set.

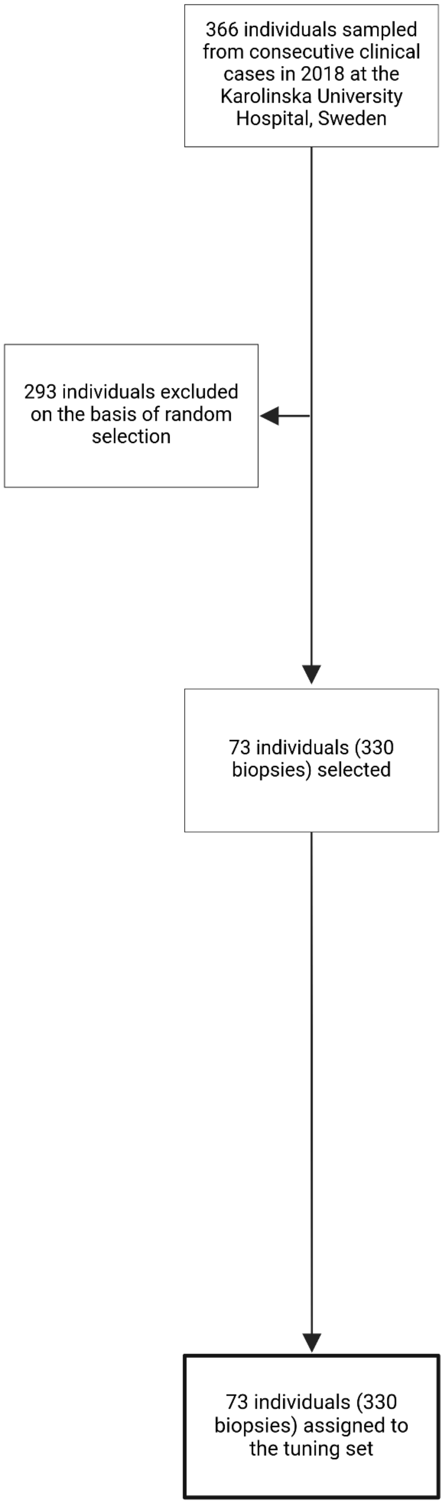

Fig S5. CONSORT diagram for the Karolinska University Hospital (KUH-1) cohort, which is part of the tuning set.

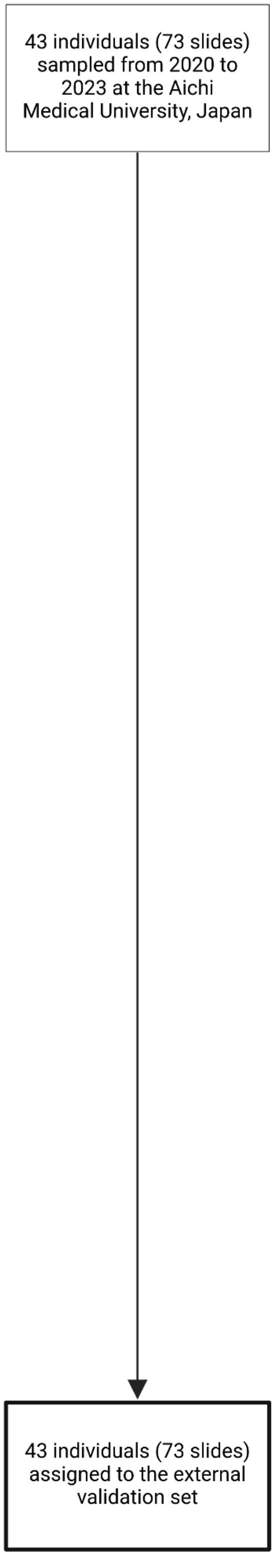

Fig S6. CONSORT diagram for the Aichi Medical University (AMU) cohort, which is part of the external validation set.

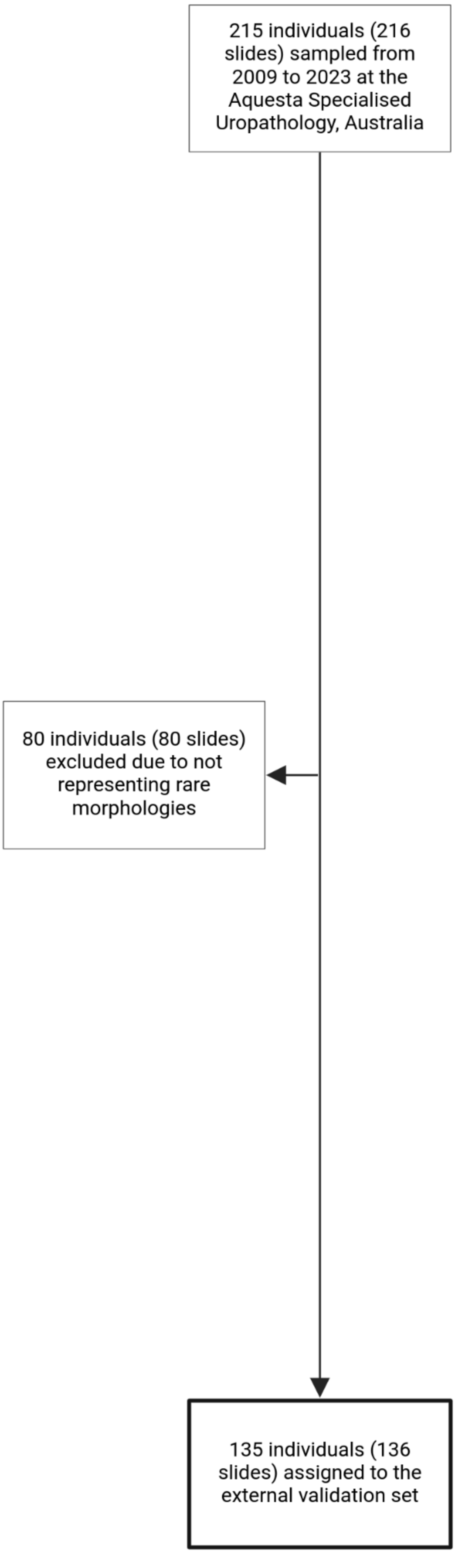

Fig S7. CONSORT diagram for the Aquesta Uropathology morphological subtypes (AQ) cohort, which is part of the external validation set.

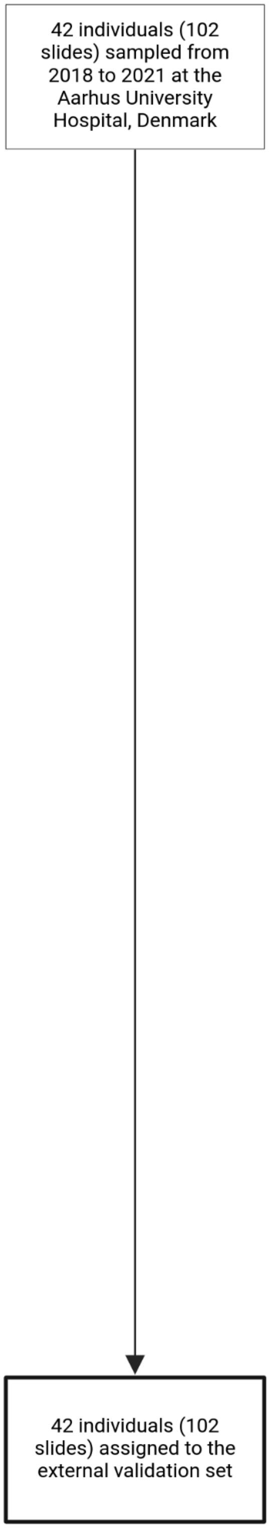

Fig S8. CONSORT diagram for the Aarhus University Hospital (AUH) cohort, which is part of the external validation set.

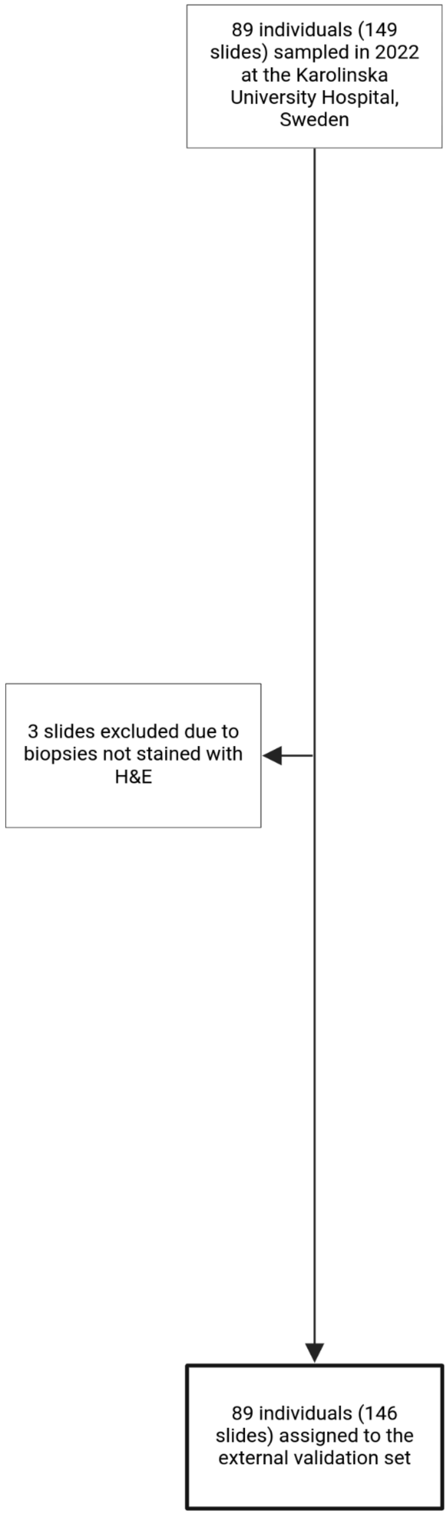

Fig S9. CONSORT diagram for the Karolinska University Hospital morphological subtypes (KUH-2) cohort, which is part of the external validation set and represents rare morphologies.

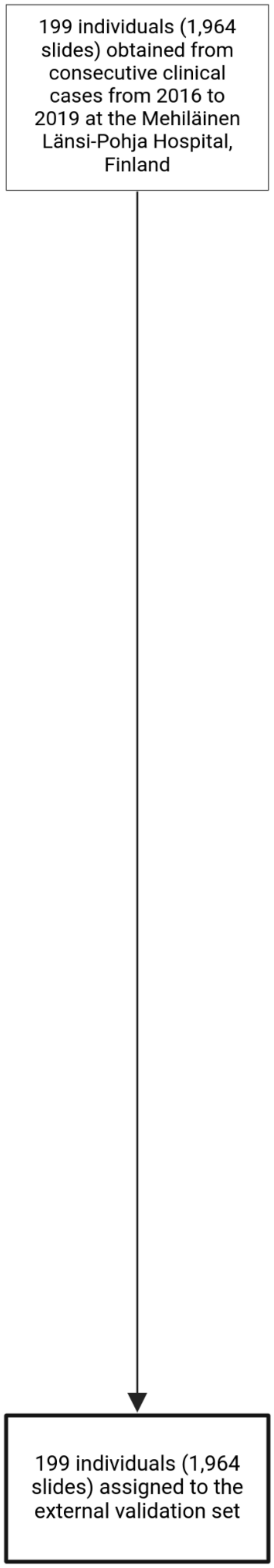

Fig S10. CONSORT diagram for the Mehiläinen Länsi-Pohja (MLP) cohort, which is part of the external validation set.

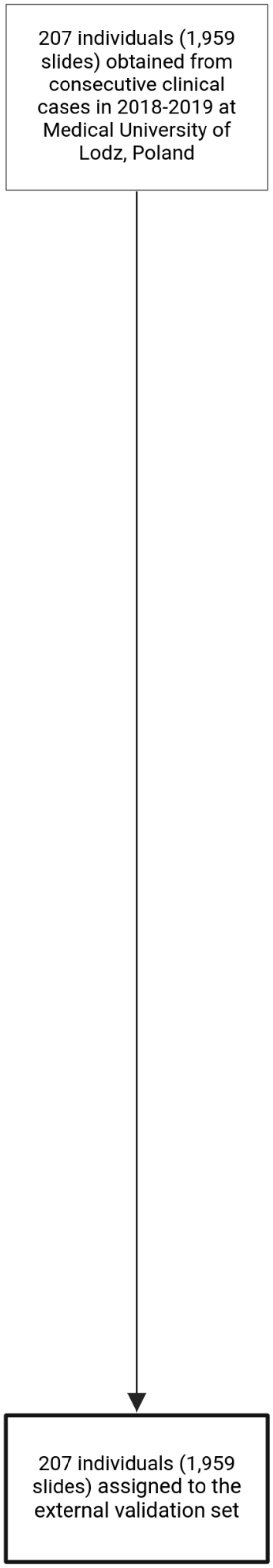

Fig S11. CONSORT diagram for the Medical University of Lodz (MUL) cohort, which is part of the external validation set.

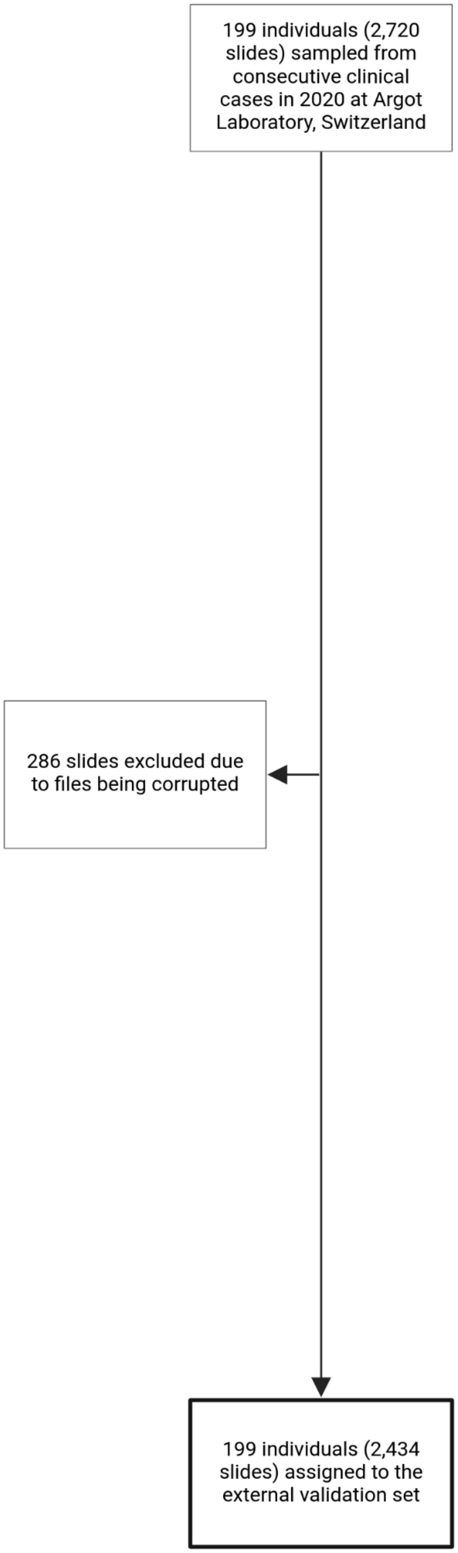

Fig S12. CONSORT diagram for the Synlab Switzerland (SCH) cohort, which is part of the external validation set.

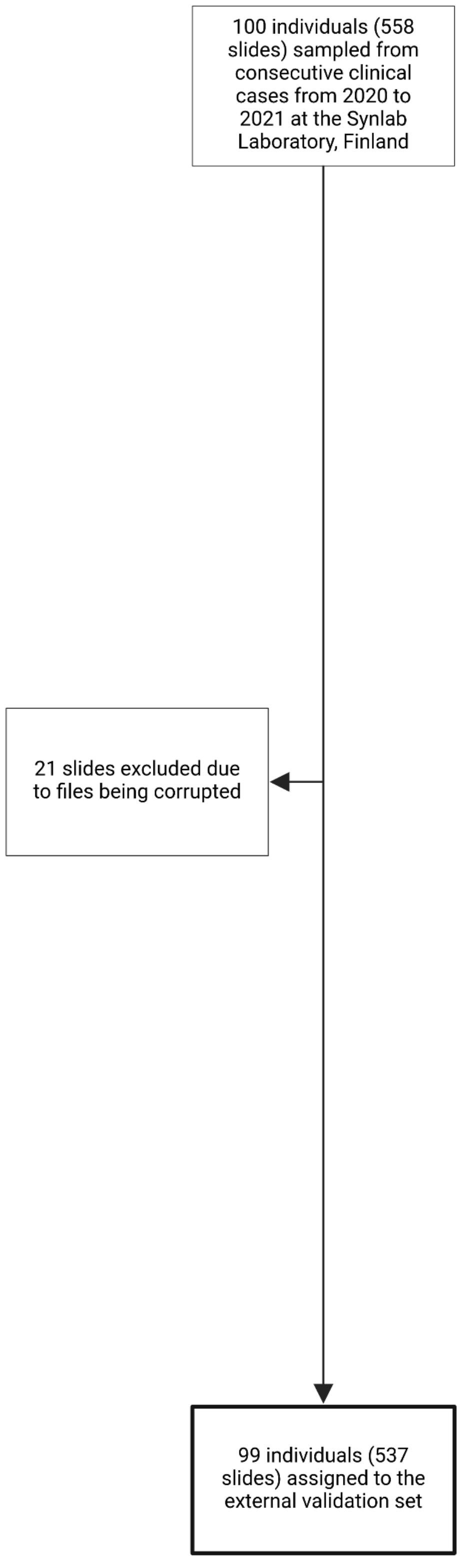

Fig S13. CONSORT diagram for the Synlab Finland (SFI) cohort, which is part of the external validation set.

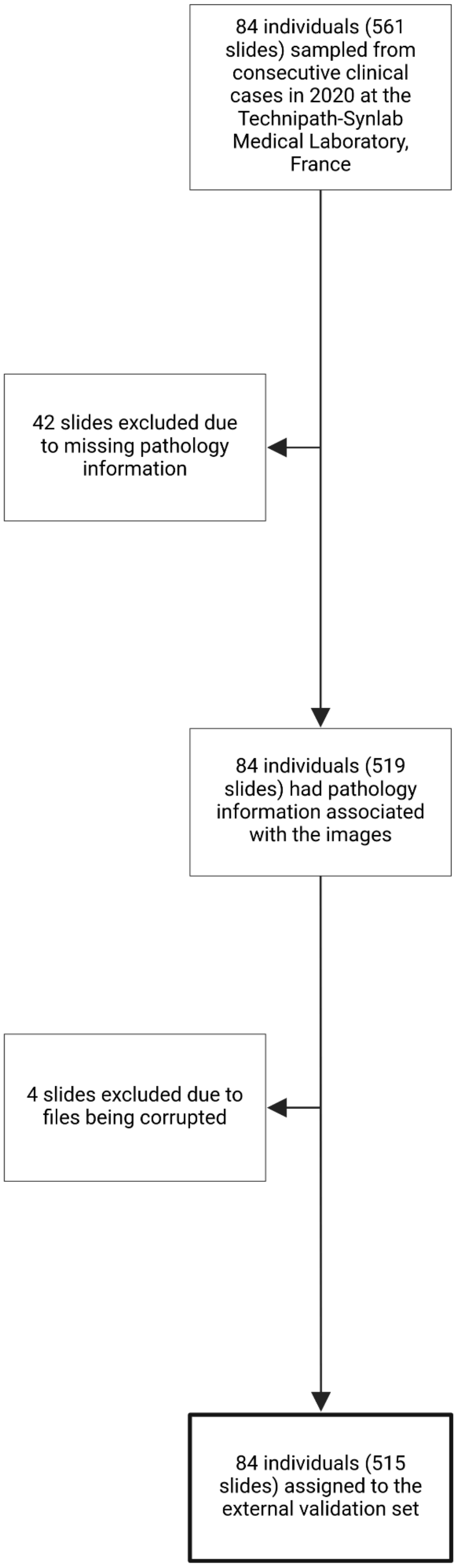

Fig S14. CONSORT diagram for the Synlab France (SFR) cohort, which is part of the external validation set.

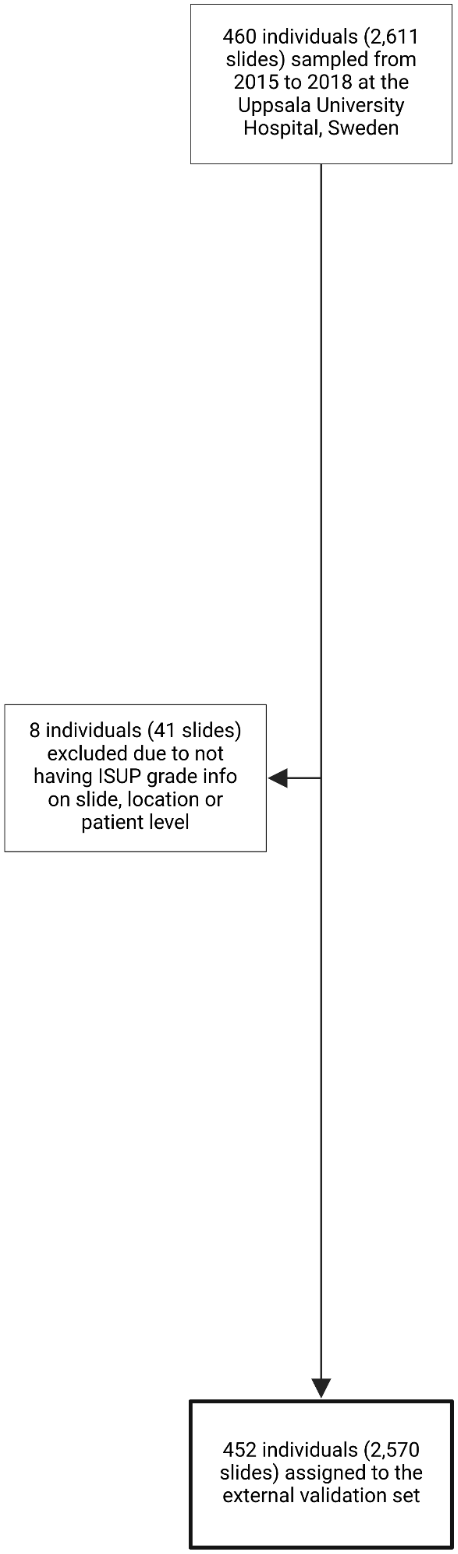

Fig S15. CONSORT diagram for the Spear Prostate Biopsy 2020 (SPROB20) cohort, which is part of the external validation set.

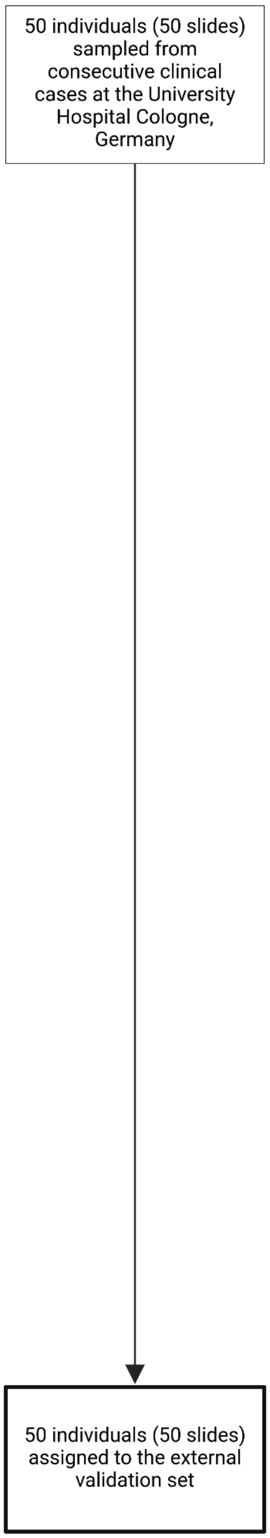

Fig S16. CONSORT diagram for the University Hospital Cologne (UKK) cohort, which is part of the external validation set.

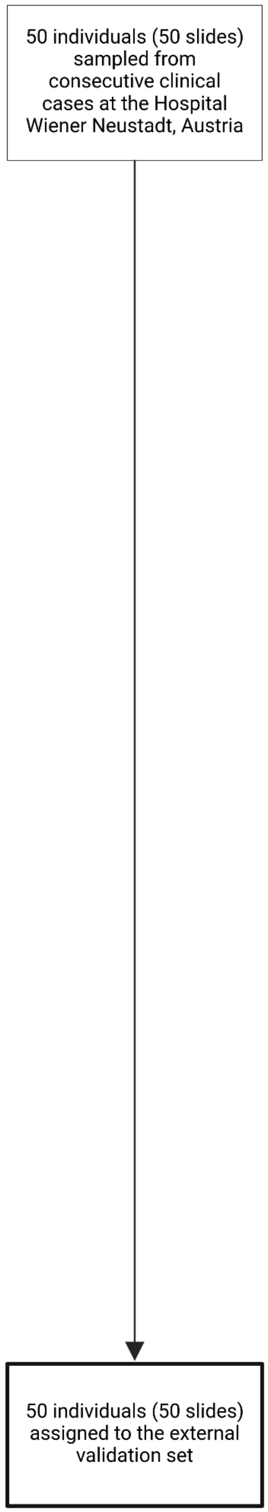

Fig S17. CONSORT diagram for the Hospital Wiener Neustadt (WNS) cohort, which is part of the external validation set.

4. REFERENCES

1. Ström P, Kartasalo K, Olsson H, Solorzano L, Delahunt B, Berney DM, et al. Artificial intelligence for diagnosis and grading of prostate cancer in biopsies: a population-based, diagnostic study. Lancet Oncol. 2020;21: 222–232.

2. Bulten W, Kartasalo K, Chen P-HC, Ström P, Pinckaers H, Nagpal K, et al. Artificial intelligence for diagnosis and Gleason grading of prostate cancer: the PANDA challenge. Nat Med. 2022;28: 154–163.

3. Bulten W, Pinckaers H, van Boven H, Vink R, de Bel T, van Ginneken B, et al. Automated deep-learning system for Gleason grading of prostate cancer using biopsies: a diagnostic study. Lancet Oncol. 2020;21: 233–241.

4. Marée R, Rollus L, Stévens B, Hoyoux R, Louppe G, Vandaele R, et al. Collaborative analysis of multi-gigapixel imaging data using Cytomine. Bioinformatics. 2016;32: 1395–1401.

5. Grönberg H, Adolfsson J, Aly M, Nordström T, Wiklund P, Brandberg Y, et al. Prostate cancer screening in men aged 50–69 years (STHLM3): a prospective population-based diagnostic study. Lancet Oncol. 2015;16: 1667–1676.

6. Kartasalo K, Ström P, Ruusuvuori P, Samaratunga H, Delahunt B, Tsuzuki T, et al. Detection of perineural invasion in prostate needle biopsies with deep neural networks. Virchows Arch. 2022;481: 73–82.

7. Olsson H, Kartasalo K, Mulliqi N, Capuccini M, Ruusuvuori P, Samaratunga H, et al. Estimating diagnostic uncertainty in artificial intelligence assisted pathology using conformal prediction. Nat Commun. 2022;13: 7761.

8. Ji X, Salmon R, Mulliqi N, Khan U, Wang Y, Blilie A, et al. Physical Color Calibration of Digital Pathology Scanners for Robust Artificial Intelligence Assisted Cancer Diagnosis. arXiv [q-bio.QM]. 2023. Available: http://arxiv.org/abs/2307.05519

9. Egevad L, Cheville J, Evans AJ, Hörnblad J, Kench JG, Kristiansen G, et al. Pathology Imagebase-a reference image database for standardization of pathology. Histopathology. 2017. pp. 677–685. doi:10.1111/his.13313

10. Egevad L, Delahunt B, Iczkowski KA, van der Kwast T, van Leenders GJLH, Leite KRM, et al. Interobserver reproducibility of cribriform cancer in prostate needle biopsies and validation of International Society of Urological Pathology criteria. Histopathology. 2023;82: 837–845.

11. Bankhead P, Loughrey MB, Fernández JA, Dombrowski Y, McArt DG, Dunne PD, et al. QuPath: Open source software for digital pathology image analysis. Sci Rep. 2017;7: 16878.

12. Egevad L, Delahunt B, Samaratunga H, Tsuzuki T, Olsson H, Ström P, et al. Interobserver reproducibility of perineural invasion of prostatic adenocarcinoma in needle biopsies.

Virchows Arch. 2021;478: 1109–1116.

13. Fredsøe J, Sandahl M, Vedsted P, Jensen JB, Ulhøi BP, Borre M, et al. Results from the PRIMA Trial: Comparison of the STHLM3 Test and Prostate-specific Antigen in General Practice for Detection of Prostate Cancer in a Biopsy-naïve Population. European Urology Oncology. 2023;6: 484–492.

14. Walhagen P, Röbeck P, Bengtsson E, Busch C, Häggman M. Spear Prostate Biopsy 2020 (SPROB20). AIDA; 2020. Available: https://datahub.aida.scilifelab.se/10.23698/aida/sprob20

15. Tolkach Y, Ovtcharov V, Pryalukhin A, Eich M-L, Gaisa NT, Braun M, et al. An international multi-institutional validation study of the algorithm for prostate cancer detection and Gleason grading. NPJ Precis Oncol. 2023;7: 77.
