# Supplementary material for: Development and retrospective validation of an artificial intelligence system for diagnostic assessment of prostate biopsies: study protocol
Source: BMJ Open. 2025 Jul 7;15(7):e097591. doi: 10.1136/bmjopen-2024-097591 (PMC12258300; doi:10.1136/bmjopen-2024-097591)
Supplement: Supplementary file 3 [file bmjopen-2024-097591.reviewer_comments.pdf]

PEER REVIEW HISTORY

BMJ Open publishes all reviews undertaken for accepted manuscripts. Reviewers are asked to complete a checklist review form and are provided with free text boxes to elaborate on their assessment. These free text comments are reproduced below.

ARTICLE DETAILS

Title (Provisional)

Study Protocol: Development and Retrospective Validation of an Artificial Intelligence System for Diagnostic Assessment of Prostate Biopsies

Authors

Mulliqi, Nita; Blilie, Anders; Ji, Xiaoyi; Szolnoky, Kelvin; Olsson, Henrik; Titus, Matteo; Martinez Gonzalez, Geraldine; Boman, Sol Erika; Valkonen, Masi; Gudlaugsson, Einar; Kjosavik, Svein Reidar; Asenjo, José; Gambacorta, Marcello; Libretti, Paolo; Braun, Marcin; Kordek, Radzislaw; Łowicki, Roman; Hotakainen, Kristina; Väre, Päivi; Bodil Ginnerup, Pedersen; Sørensen, Karina Dalsgaard; Ulhøi, Benedicte Parm; Rantalainen, Mattias; Ruusuvuori, Pekka; Delahunt, Brett; Samaratunga, Hema; Tsuzuki, Toyonori; Janssen, Emilius Adrianus Maria; Egevad, Lars; Kartasalo, Kimmo; Eklund, Martin

---

VERSION 1 - REVIEW

**Reviewer**	**1**
**Name**	**Bauman, Glenn**
**Affiliation**	**Western University, London Regional Cancer Program**
**Date**	**02-Feb-2025**
**COI**	**None**

This study protocol meticulously documents a prospective approach to developing AI classifiers for key pathologic findings in prostate cancer biopsies. Key guidance for developing such tools are followed and referenced. There is a rigorous "accounting" of the cohorts to be used and how they will be split between training, tuning and internal and external validation. There are only a few areas where clarifications could help:

1. for classifying cribiform pattern, why was IDC not included as well? Or is cribiform and IDC being considered together. Please clarify as they are both independent adverse predictors (i.e.doi.org/10.1016/j.eururo.2025.01.013)

2. In table 1. there are blank columns between cohorts regarding missing data. I couldn't find an accounting for the reasons for the missing data (but I'll admit I didn't scour the

supplementary data) A notation in the Table legend as to where to find this detail if it is there would be helpful...or add it if it is missing)

3. Figure 1. nicely shows the different cohorts for stages of the work. I assume the different colours for the icons for the cohorts refer to specific institution/data sets and if this is the case this should be outlined in the Figure legend or as a footnote to the figure.

Otherwise, this seems to me a "best in class" effort to develop a rigorous, clinically useful (and ultimately clinically acceptable) deep learning/machine learning model for an important classification task.

Reviewer **2**

Name **Telecan, Teodora**

Affiliation **Iuliu Hagieganu University of Medicine and Pharmacy Faculty of Medicine**

Date **22-Apr-2025**

COI **None**

Overall, the paper is well put together, with clear and proper use of the English language. The proposed protocol is thorough, with clear aims and guidelines for future publications. Therefore, I have only minor mentions to make:

- Consider adding epidemiological data that highlight the importance of this malignancy for the male population and its impact on the healthcare systems.

- I suggest providing more exact percentages regarding the interobserver variability in terms of Gleason score grading, the accuracy of AI diagnostic accuracy and accuracy variation when external-source WSI were analyzed by these studies.

- How many cores were sampled per patient? Were the biopsies systematic or targeted?

- Were there exclusion criteria based on the experience level of the urologist/radiologist who performed the biopsies?

VERSION 1 - AUTHOR RESPONSE

Reviewer 1: This study protocol meticulously documents a prospective approach to developing AI classifiers for key pathologic findings in prostate cancer biopsies. Key guidance for developing such tools are followed and referenced. There is a rigorous "accounting" of the cohorts to be used and how they will be split between training, tuning and internal and external validation. There are only a few areas where clarifications could help:

1. for classifying cribiform pattern, why was IDC not included as well? Or is cribiform and IDC being considered together. Please clarify as they are both independent adverse predictors (i.e.doi.org/10.1016/j.eururo.2025.01.013)

We thank the reviewer for this insightful comment. For our secondary study objective of detecting cribriform cancer, the AI model is being developed and validated to identify cribriform growth pattern irrespective of whether it occurs as part of acinar adenocarcinoma (i.e., invasive cribriform) or intraductal carcinoma (IDC) (i.e., non-invasive cribriform). The rationale behind this approach is that in diagnostic practice, many pathologists tend to pool these patterns together to decide on treatment and prognostication. The exception is when possible IDC is encountered without any evidence of concomitant invasive cancer - in this scenario, many pathologists agree that the IDC component should not be graded, but rather given a comment stating the usual association of IDC with aggressive invasive cancer. This scenario is rarely encountered (0.06-0.26% of biopsies) [1], and in most of these cases, there will be unsampled invasive carcinoma present elsewhere in the prostate [1]. In such cases where exclusive IDC is a possibility, IHC is usually performed by the investigating pathologist to determine whether there exists an invasive cribriform component justifying Gleason grading. However, in the vast majority of cases, i.e. when cribriform morphology is seen together with definitive invasive cancer, pooling cribriform-IDC with invasive-cribriform cancer streamlines the diagnostic workflow and reduces the need for additional immunohistochemistry. This practice is supported by the 2019 ISUP consensus [1], which recommended incorporating IDC into the Gleason grading when present alongside invasive carcinoma. However, we acknowledge the fact that both these are independent adverse predictors, and in some cases (even though rare), IDC could be present without the invasive carcinoma. We have clarified these motives in the revised manuscript under the "Study design" section.

[1] van Leenders GJLH, van der Kwast TH, Grignon DJ, Evans AJ, Kristiansen G, Kweldam CF, Litjens G, McKenney JK, Melamed J, Mottet N, Paner GP, Samaratunga H, Schoots IG, Simko JP, Tsuzuki T, Varma M, Warren AY, Wheeler TM, Williamson SR, Iczkowski KA; ISUP Grading Workshop Panel Members. The 2019 International Society of Urological Pathology (ISUP) Consensus Conference on Grading of Prostatic Carcinoma. Am J Surg Pathol. 2020 Aug;44(8):e87-e99. doi: 10.1097/PAS.0000000000001497. PMID: 32459716; PMCID: PMC7382533.

2. In table 1, there are blank columns between cohorts regarding missing data. I couldn't find an accounting for the reasons for the missing data (but I'll admit I didn't scour the supplementary data) A notation in the Table legend as to where to find this detail if it is there would be helpful...or add it if it is missing)

We have updated the legends of Table 1 (and Table 4) to clarify that the blank columns in the table (now marked as “N/A”) represent missing information in our database. This information could not be retrieved due to differences in data collection across sites and cohorts. Our data collection occurred in multiple rounds and ways, including structured databases, pathology reports, and simple pre-filled spreadsheets provided by site-specific pathologists. In cases where detailed pathology reports were accessible, we could retrieve comprehensive clinical data, and in other cases, pathologists simply did not report this information at the time of data submission.

3. Figure 1. nicely shows the different cohorts for stages of the work. I assume the different colours for the icons for the cohorts refer to specific institution/data sets and if this is the case this should be outlined in the Figure legend or as a footnote to the figure.

The different icon colours in Figure 1 indeed indicate separate unique patient cohorts within the data partitions. That is, the same color is for example used to indicate the same cohort appearing in the training and internal validation partitions. We agree that this is something that could be mentioned and would further assist the reader. We have emphasised this in the caption of Figure 1 in the revised version of the manuscript.

Otherwise, this seems to me a "best in class" effort to develop a rigorous, clinically useful (and ultimately clinically acceptable) deep learning/machine learning model for an important classification task.

We thank the reviewer for these very kind words!

Reviewer 2: Overall, the paper is well put together, with clear and proper use of the English language. The proposed protocol is thorough, with clear aims and guidelines for future publications. Therefore, I have only minor mentions to make:

- Consider adding epidemiological data that highlight the importance of this malignancy for the male population and its impact on the healthcare systems.

We thank the reviewer for this thoughtful suggestion. We fully agree that emphasising the epidemiological aspect of prostate cancer adds important context to the motivation for this study. In response, we have revised the "Introduction" section to highlight prostate cancer incidence and its associated diagnostic demands. We believe this addition further strengthens the rationale for developing AI-based tools to support pathologists in diagnostic workflows.

- I suggest providing more exact percentages regarding the interobserver variability in terms of Gleason score grading, the accuracy of AI diagnostic accuracy, and accuracy variation when external-source WSI were analyzed by these studies.

We agree that it would be beneficial to highlight the interobserver variability and AI performance accuracy in relation to validation data with concrete measures. In the revised "Introduction" section of the manuscript, we mention the span of agreement rates amongst pathologists measured by kappa statistics, as reported in prior literature. However, comprehensive comparison of previously reported accuracies of AI systems for this task on internal and external data would necessitate a careful meta-analysis approach. The value of the kappa statistic, for example, depends on the composition of the validation dataset it is estimated on (e.g. Gleason score distribution), which complicates direct comparisons between studies that have relied on different datasets. Such a meta-analysis is outside the scope of this study protocol, and we therefore prefer not to cite isolated statistical results from prior studies, which may be prone to misinterpretation.

- How many cores were sampled per patient? Were the biopsies systematic or targeted?

The biopsy sampling across cohorts involved systematic or targeted biopsies or a mix of the two. The number of cores per patient depends on the sampling method, with systematic typically having 10 to 12 cores, and the targeted approach collecting up to 6 cores per patient. We do not have information on the exact distribution of the targeted vs systematic biopsies in cohorts where both of the sampling techniques were used. The information on biopsy sampling is currently listed under "Data cohorts" in the Supplementary Appendix 2. However, in the

revised version of the manuscript, in the “Study design” section, we explicitly refer the reader to the supplementary appendix file for this information.

- Were there exclusion criteria based on the experience level of the urologist/radiologist who performed the biopsies?

There were no exclusion criteria in place based on the experience level of the clinician performing the biopsy. The full set of criteria can be found in the “Inclusion and exclusion criteria“ section.
